# Comparison of the effect of treatment with NSAIDs added to anti-TNF therapy versus anti-TNF therapy alone on the progression of structural damage in the spine over 2 years in patients with radiographic axial spondyloarthritis from the randomised-controlled CONSUL trial

**DOI:** 10.1136/ard-2023-224699

**Published:** 2024-01-16

**Authors:** Fabian Proft, Murat Torgutalp, Burkhard Muche, Valeria Rios Rodriguez, Joachim Listing, Mikhail Protopopov, Judith Rademacher, Hildrun Haibel, Laura Spiller, Anne-Katrin Weber, Maryna Verba, Jan Brandt-Juergens, Uta Kiltz, Maren Sieburg, Swen Jacki, Joachim Sieper, Denis Poddubnyy

**Affiliations:** 1 Department of Gastroenterology, Infectiology and Rheumatology (including Nutrition Medicine), Charite Universitatsmedizin Berlin, Berlin, Germany; 2 Department of Rheumatology CCM, Charite Universitatsmedizin Berlin, Berlin, Germany; 3 German Rheumatism Research Center Berlin, Berlin, Germany; 4 BIH, Berlin, Germany; 5 Rheumatology, Private Practice, Berlin, Germany; 6 Rheumazentrum Ruhrgebiet, Herne, Germany; 7 Rheumatologische Facharztpraxis, Magdeburg, Germany; 8 Rheumatologische Schwerpunktpraxis, Tübingen, Germany

**Keywords:** Anti-Inflammatory Agents, Non-Steroidal, Biological Therapy, Spondylitis, Ankylosing, Tumor Necrosis Factor Inhibitors

## Abstract

**Objectives:**

The study aimed to evaluate the effect of adding a non-steroidal anti-inflammatory drug (NSAID), celecoxib (CEL), to a tumour necrosis factor inhibitor (TNFi), golimumab (GOL), compared with TNFi monotherapy on radiographic spinal progression in patients with radiographic axial spondyloarthritis (r-axSpA) over 2 years.

**Methods:**

R-axSpA patients, having risk factors for radiographic progression (high disease activity plus C reactive protein >5 mg/L and/or ≥1 syndesmophyte(s)), underwent a 12-week run-in phase with GOL 50 mg every 4 weeks. In the core phase (96 weeks), only patients with a good clinical response at week 12 were randomised (1:1) to GOL+CEL 200 mg two times per day (combination therapy) or GOL monotherapy. The primary endpoint was radiographic progression assessed by modified Stoke Ankylosing Spondylitis Spinal Score (mSASSS) change at week 108 in the intent-to-treat population.

**Results:**

A total of 128 patients were enrolled in the run-in phase; and 109 patients were randomised at week 12 to monotherapy (n=55) or combination therapy (n=54). At week 108, 97 (52 vs 45) patients completed the study. The change in mSASSS at week 108 was 1.7 (95% CI 0.8 to 2.6) in the monotherapy vs 1.1 (95% CI 0.4 to 1.8) in the combination therapy groups (p=0.79). New syndesmophytes occurred in 25% of patients in the monotherapy vs 11% of patients in the combination therapy groups (p=0.12). During the study, no significant differences in adverse events and serious adverse events were observed between the groups.

**Conclusions:**

Combination therapy with GOL+CEL did not demonstrate statistically significant superiority over GOL monotherapy in retarding radiographic spinal progression over 2 years in r-axSpA.

**WHAT IS ALREADY KNOWN ON THIS TOPIC**

- Continuous intake of celecoxib over 2 years was associated with retardation of radiographic spinal progression in a previous study in radiographic axial spondyloarthritis (r-axSpA); this effect was most pronounced in patients with elevated C reactive protein.
- However, such an effect was not observed in another trial comparing continuous and on-demand treatment with the non-selective cyclooxygenase inhibitor diclofenac over 2 years in r-axSpA. Tumour necrosis factor inhibitors (TNFis) also indicated an ability to retard radiographic spinal progression; however, this effect was not seen until at least 4 years after starting treatment.
- The effect of combination therapy in high-risk patients has not been investigated so far.

**WHAT THIS STUDY ADDS**

- This is the first prospective, randomised-controlled trial to investigate the effect of continuous intake of a non-steroidal anti-inflammatory drug (NSAID) (celecoxib) in combination with a TNF inhibitor (golimumab) in patients with r-axSpA at high risk of radiographic spinal progression.
- The combination of celecoxib and golimumab did not show statistically significant superiority over golimumab monotherapy in retarding radiographic spinal progression over 2 years in patients with r-axSpA.
- However, the observed numerical difference in favour of combination therapy may be relevant for high-risk patients in situations where combination therapy is clinically indicated.

**HOW THIS STUDY MIGHT AFFECT RESEARCH, PRACTICE OR POLICY**

- Based on these data, a continuous treatment with NSAIDs to reduce radiographic spinal progression in r-axSpA patients with otherwise good disease activity control cannot be routinely recommended.
- However, the observed numerical difference in favour of combination therapy may be relevant for high-risk patients in situations where combination therapy is clinically indicated and in patients showing signs of remaining disease activity despite bDMARD/anti-TNF therapy, combination with an NSAID (celecoxib) may be considered to add a potentially retarding effect on radiographic progression based on the data from this study.

## Introduction

Axial spondyloarthritis (axSpA) is a chronic inflammatory immune-mediated disease characterised by inflammation preferentially of the axial skeleton—the sacroiliac joints and the spine.^1^ Under the umbrella term ‘axSpA’, two forms (or stages) of the disease are included: non-radiographic axial spondyloarthritis (nr-axSpA)—without structural damage in the sacroiliac joints visible on radiographs, and radiographic axial spondyloarthritis (r-axSpA; also known as ankylosing spondylitis (AS))—with definite radiographic sacroiliitis fulfilling the radiographic criterion of the modified New York criteria.^2^ Although the distinction between nr-axSpA and r-axSpA does not play a meaningful clinical role, it is still relevant for research purposes. In addition, structural damage in the spine that is mostly attributable to new bone formation, the development of so-called ‘syndesmophytes’ and ankyloses in the spine is usually seen at a later, radiographic stage of the disease. Structural damage in the spine, along with disease activity, is one of the two foremost determinants of the physical function and spinal mobility (and assessed by the Bath Ankylosing Spondylitis Functional Index (BASFI)^3^ and by the Bath Ankylosing Spondylitis Metrology Index (BASMI)^4^ in patients with axSpA over the long term.^5 6^

The current standard to quantify structural damage in the spine associated with the disease progression is the modified Stoke Ankylosing Spondylitis Spinal Score (mSASSS),^7^ which allows for evaluation of damage visible on plain radiographs (and, therefore, referred to as radiographic spinal progression) of the cervical and lumbar spine.^8^ Previous research has identified a number of factors associated with radiographic spinal progression, including pre-existing structural damage (syndesmophytes), high disease activity as reflected by elevated C reactive protein (CRP), Axial Spondyloarthritis Disease Activity Score (ASDAS) and by the presence of inflammation on MRI.^9–12^

Previous data suggest that non-steroidal anti-inflammatory drugs (NSAIDs), in particular the selective cyclooxygenase (COX)-II inhibitor celecoxib, may have disease-modifying properties in r-axSpA in addition to their anti-inflammatory and symptomatic effects, as shown in a long-term extension of a randomised-controlled trial (RCT).^13^ The afore-mentioned retarding effect of celecoxib on the spinal structural changes in r-axSpA patients was particularly evident in patients with elevated CRP levels.^14^ However, such an effect was not observed for the non-selective COX inhibitor diclofenac in a recent RCT.^15^ Notably, the results from the GErman Spondyloarthritis Inception Cohort (GESPIC) demonstrated that higher NSAID intake (defined as >50% of the maximum dose) may retard radiographic spinal progression. This effect was most pronounced in r-axSpA patients who were at high risk of radiographic spinal progression (due to the presence of syndesmophytes and/or elevated CRP at baseline).^16^

For tumour necrosis factor inhibitors (TNFis), which are usually used in patients with axSpA, who do not respond to or cannot tolerate NSAIDs, most of the trials did not show an inhibitory effect on radiographic spinal progression during the first 2 years of treatment.^17–19^ However, observational data indicated such an effect for TNFi after a longer treatment period.^20–26^

Whether the addition of an NSAID (particularly a COX-II selective one) to a TNFi therapy in patients with r-axSpA might have a better inhibitory effect on radiographic spinal progression in the first 2 years of treatment as compared with TNFi therapy alone has not been investigated so far.

In the ‘COmparison of the effect of treatment with NSAIDs added to anti-TNF therapy versus anti-TNF therapy alone on progression of structural damage in the spine over two years in patients with ankyLosing spondylitis—CONSUL’ study, we aimed to assess the effect of adding celecoxib to golimumab compared with golimumab monotherapy on radiographic spinal progression over 2 years in r-axSpA patients at high risk (either presence of syndesmophytes and/or elevated CRP) of radiographic spinal progression.

## Methods

### Study design

**Figure 1:**
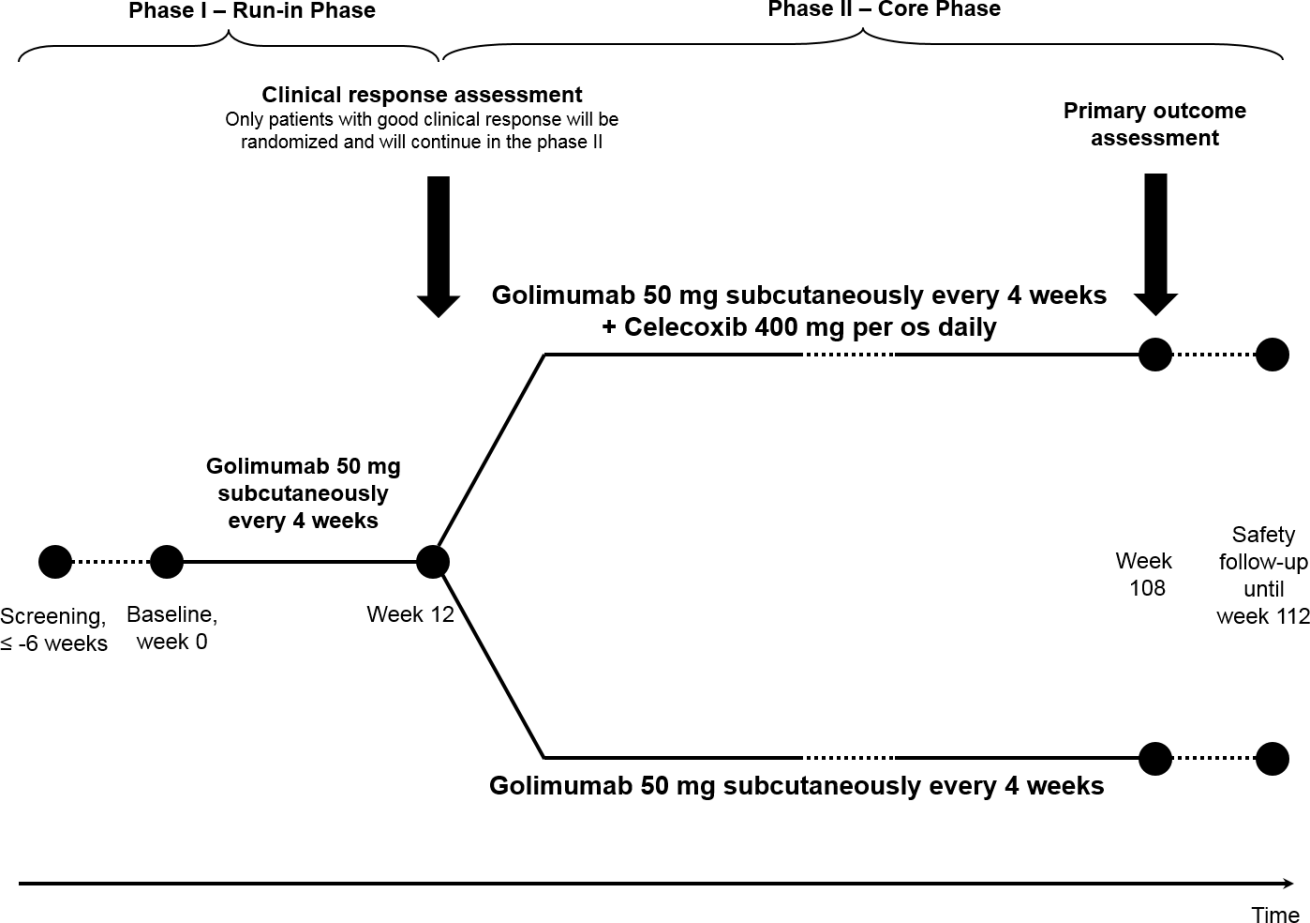
Study design of the COmparison of the effect of treatment with NSAIDs added to anti-TNF therapy versus anti-TNF therapy alone on progression of structural damage in the spine over two years in patients with ankyLosing spondylitis study.

The CONSUL study (figure 1) was a randomised, controlled, multicentre, open-label clinical phase IV trial. The study consisted of a 6-week screening period, a 12-week period (phase I: ‘run-in phase’) of treatment with golimumab 50 mg subcutaneously (s.c.) every 4 weeks for all subjects followed by a 96-week controlled treatment period (phase II: ‘core phase’) with golimumab plus celecoxib versus golimumab alone for patients with a sufficient treatment response to golimumab, and a safety follow-up period of 4 weeks (https://bmjopen.bmj.com/content/bmjopen/7/6/e014591.full.pdf).^27^ Only those patients with a good clinical response to golimumab in phase I (improvement of the Bath Ankylosing Spondylitis Disease Activity Index (BASDAI) Score by ≥2 absolute points on a 0–10 numerical rating scale (NRS) at week 12^28^ were eligible for phase II and were randomised based on a 1:1 ratio to receive golimumab 50 mg s.c. every 4 weeks plus celecoxib (in a daily dose of 400 mg/day) (=combination therapy) or golimumab 50 mg s.c. every 4 weeks alone (=monotherapy) for another 96 weeks followed by a 4-week safety follow-up.^27^ The details of the study protoco and of the randomisation procedure can be found as online supplemental file 2.

10.1136/ard-2023-224699.supp2 Supplementary data

### Patients and assessments

We recruited adult patients with the clinical diagnosis of r-axSpA fulfilling the radiographic criterion of the modified New York criteria for ankylosing spondylitis, with active disease (BASDAI≥4/10), who failed first-line treatment (NSAIDs) according to the international management recommendations, and who had at least one of the two following risk factors for radiographic spinal progression: an elevated CRP (>5 mg/L) at screening and/or existing structural damage (≥1 syndesmophyte(s) in the spine on plain radiography obtained at screening or in the previous 24 months as judged by the investigator).^27^ If the patient was currently treated with an NSAID, other analgesic or the conventional synthetic disease-modifying antirheumatic drugs (DMARDs) methotrexate or sulfasalazine, the dose must have been stable for at least 2 weeks prior to the baseline visit. Patients could have a history of previous biological DMARD (bDMARD) treatment, if no history of primary non-response to TNFi was documented and if the previous bDMARD treatment was discontinued at least 12 weeks prior to the baseline visit.^27^ The cumulative dose of an NSAID in the previous 3 months at each visit was calculated using the NSAID intake score as recommended by ASAS.^29^ Key exclusion criteria were total spinal ankylosis and contraindications for the treatment with golimumab and/or celecoxib (table 1). A detailed list of the inclusion and exclusion criteria can be found in the study protocol as online supplemental file 1.

10.1136/ard-2023-224699.supp1 Supplementary data

**Table 1:** Key inclusion and exclusion criteria of the CONSUL study

Inclusion criteria	Exclusion criteria
Age≥18 years	Presence of total spinal ankylosis
Definite diagnosis of AS according to the modified New York criteria	History of primary non-response to previous anti-TNF therapy (if any)
Active disease (defined as BASDAI≥4)	Contraindications for the treatment with golimumab and/or celecoxib
History of an inadequate response to therapeutic trials of at least two NSAIDs	
≥1 risk factor for radiographic spinal progression (defined as elevated CRP and/or existing syndesmophyte(s) at screening)	

AS, ankylosing spondylitis; BASDAI, Bath Ankylosing Spondylitis Disease Activity Index; CONSUL, COmparison of the effect of treatment with NSAIDs added to anti-TNF therapy versus anti-TNF therapy alone on progression of structural damage in the spine over two years in patients with ankyLosing spondylitis; CRP, C reactive protein; NSAIDs, non-steroidal anti-inflammatory drugs; TNF, tumour necrosis factor.

In order to clearly differentiate the NSAID intake after randomisation between the two groups, all patients in the combination therapy group were allowed to reduce celecoxib dose to a minimum of 200 mg per day in case of intolerability or occurrence of adverse events (AEs). Celecoxib intake was prohibited in the monotherapy group with golimumab 50 mg s.c. every 4 weeks, and study physicians were encouraged to avoid NSAID prescription where possible. If NSAID treatment was needed, it was only allowed up to 50% of the maximal daily dosage of the respective NSAID and for the shortest time possible.

### Outcomes

The primary endpoint of the study was defined as change in radiographic spinal progression measured by the mSASSS^30^ over 2 years of treatment. For this assessment, radiographs of the cervical and lumbar spine in lateral view were performed locally at screening (or already available radiographs performed within 24 months before screening) and after 2 years of treatment (week 108 per protocol, week 84 at the earliest for patients who discontinued prematurely). The mean duration between the radiographs and the baseline visit for the patients in the combination therapy group was 0.89 months (95% CI: 0.37 to 1.41), while this was 1.16 months (95% CI: 0.30 to 2.03) for the monotherapy group (p=0.76). Images were centrally stored in a digital and anonymised format and subsequently scored independently by three trained and calibrated readers (FP, MT and VRR), who were blinded for treatment group allocation, the time point of the assessment and all clinical data.

Secondary endpoints included new syndesmophyte formation or progression of existing syndesmophytes, improvement in disease activity, physical function, spinal mobility and health-related quality of life according to the relevant disease outcome measures including ASDAS (CRP based), BASDAI, CRP, BASFI, BASMI, ASAS Health Index, patient global assessment of disease activity and spinal and nocturnal pain on an NRS of 0–10. Safety endpoints included the number of AEs and percentage of patients experienced AE, serious AE (SAE) and events of interest: infections, malignancies, gastrointestinal events (ulceration, bleeding, perforation, gastric outlet obstruction), thrombotic cardiovascular events (myocardial infarction, stroke, pulmonary artery embolism, peripheral arterial of venous thrombosis), renal impairment and hepatic deterioration. The full assessment schedule can be found in the study protocol (online supplemental file 2).

### Sample size calculation

The sample size calculation was based on the findings of Kroon *et al*,^14^ data from the GESPIC Cohort^16^ and radiographic spinal progression data from the phase III golimumab study.^31^ We assumed a worsening in the mSASSS Score of 1.7±2.8 in patients of the golimumab monotherapy group and of 0.2±1.6 in patients receiving a combination of golimumab with celecoxib. Such a difference of 1.5 mSASSS units has also been observed for on-demand celecoxib use in a previous RCT not enriched for risk factors for radiographic progression.^13^ If our study design would overlook a more subtle treatment effect, such an outcome would not be deemed relevant. To detect the anticipated difference with an 80% power (using a two-sided Welch-Satterthwaite t-test, α=0.05), the sample size of n=38 in each group was deemed necessary in core phase of the trail. This is also true if the Mann-Whitney test is applied.

### Statistical analysis

The statistical evaluation of the safety and efficacy was conducted according to the intention-to-treat (ITT) principle. The analyses were based on all patients randomised who received at least one dose of the study drug in the core phase of the study.

The change in the mSASSS over a period of 2 years (Δ mSASSS=mSASSS study end – mSASSS study entry) was used as primary efficacy endpoint. The mean of the mSASSS scores of the three readers was used to calculate the final score at each time points.

The mSASSS Score was calculated only if not more than eight vertebral edges score were missing per time point. In this case, single missing score was replaced by the values of the second time point or by 0 if both time points were missing. Multiple imputation methods were used to replace missing mSASSS change scores if the radiograph was completely missing or if the number of missing vertebral edges score exceeded 8. Imputation was done separately for both treatment groups and separately for the data of each of the three readers by means of the predictive mean method of the SAS PROC MI procedure. The number of imputations was 20. The following covariables were incorporated in the imputation model: the log-transformed mSASSS Score at baseline, the time lag between screening and the ‘baseline radiographic assessment’ (≤3 months, >3 to 6 months, >6 months) and the log-transformed mean CRP values under treatment.

The statistical analysis of the primary endpoint was based on the two-sided Mann-Whitney U test statistic with a two-sided type I error rate α=0.05. The results of the corresponding rank tests of the 20 imputed datasets were combined and pooled to a final p value according to Rubin’s method by means of SAS PROC MIANALYZE. Probability plots were used to depict radiographic spinal progression. In the analysis of syndesmophytes, new syndesmophytes (change of the individual corner score from 0 or 1 to 2 or 3) and progression of syndesmophytes (development of a bridge out of two single syndesmophytes as indicated by the progression from the individual corner scores from 2 to 3) were accounted only if scored by all three readers. Fisher’s exact test was applied for the intergroup comparisons.

To further investigate the change in mSASSS between the two treatment groups, we performed the following sensitivity analyses: (1) an analysis of covariance (ANCOVA) adjusted for baseline mSASSS and the time lag between screening and the time of the first (baseline) image as well as for prior exposure to bDMARDs; (2) an analysis restricted to patients who completed the study according to the protocol and had complete sets of radiographs at both time points; (3) an analysis adjusting for NSAID use in the core phase of the study (excluding patients in the combination therapy group with an NSAID intake index<80 and patients in the monotherapy group with an NSAID index>20); (4) an analysis of patients at higher risk of progression at baseline, defined as: (a) patients with an elevated CRP, (b) patients with at least one syndesmophyte at baseline and (c) patients with an elevated CRP and syndesmophyte(s) at baseline and (5) a linear mixed effects model to evaluate the time course of mSASSS between groups (for this analysis, a time–treatment interaction term and baseline mSASSS were added into the model).

Mixed linear models for repeated measures were used to analyse the mean course of disease activity, function and global health in the core study phase between week 12 and week 108.

The inter-reader variability was evaluated by the calculation of the intraclass correlation coefficients (ICC) for the status and for the change scores.

### Patient and public involvement statement

Patients and the public were not involved in the design, conduct, reporting or dissemination plans of this study.

## Results

### Demographic and clinical characteristics

**Figure 2:**
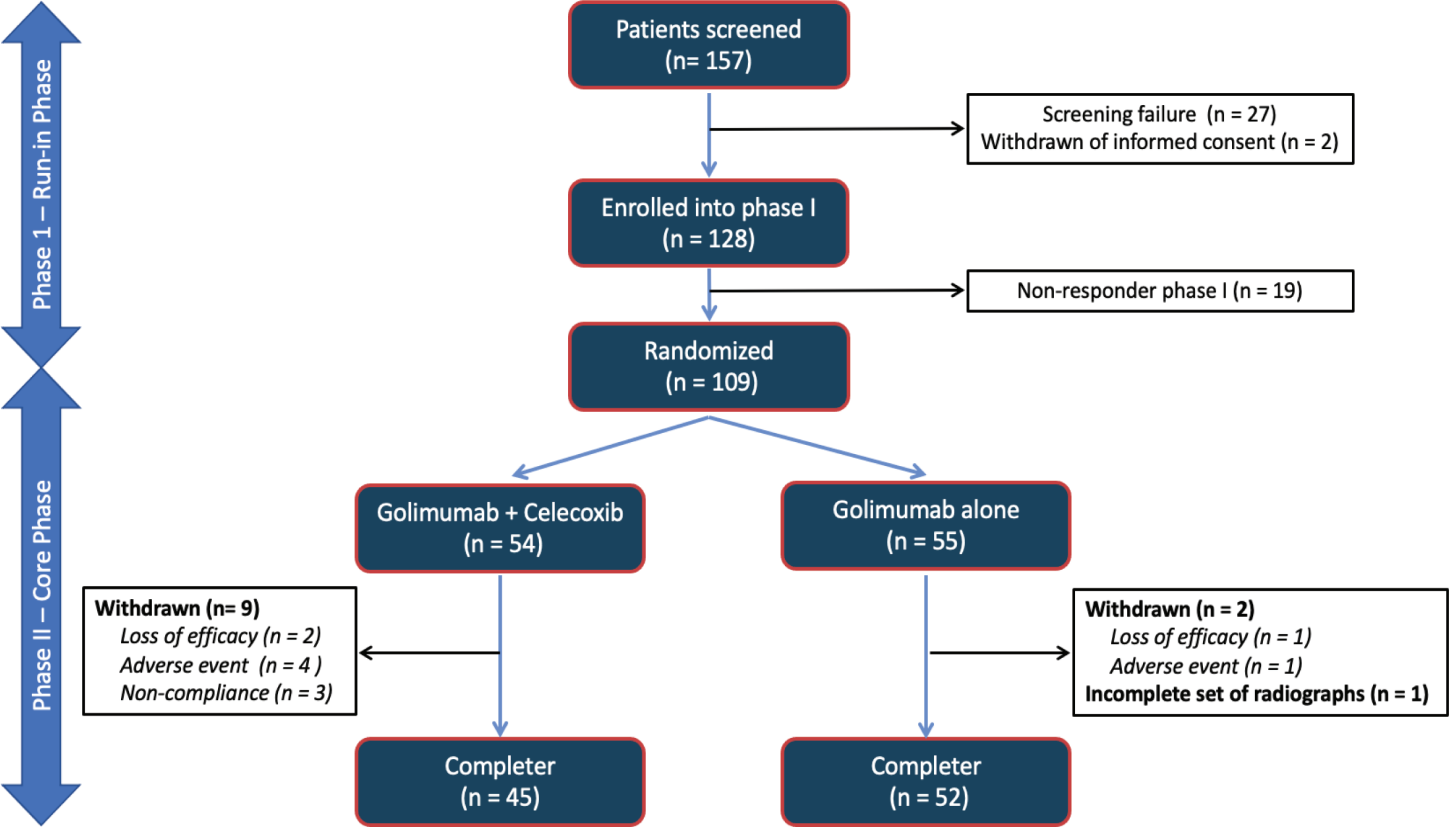
Flow chart of the COmparison of the effect of treatment with NSAIDs added to anti-TNF therapy versus anti-TNF therapy alone on progression of structural damage in the spine over two years in patients with ankyLosing spondylitis study.

In total, 128 of the 157 screened patients (81.5%) were enrolled at 16 centres from Germany between September 2016 and January 2019 into the run-in phase of the study. At week 12, 109 patients fulfilled the BASDAI response criterion and were randomised on a 1:1 basis to the combination therapy with golimumab+celecoxib or with golimumab monotherapy (54 in the combination therapy arm and 55 in the monotherapy group). Of those 109 patients, 97 patients (89%) completed the study at week 108, with 45 patients (83.3%) in the combination therapy group and 52 patients (94.5%) in the monotherapy arm (figure 2).

Baseline characteristics of the 109 randomised patients were in general similar between both groups and are shown in detail in table 2: patients were predominantly male (74.3%) and HLA-B27 positive (87.6%) with a mean age of 38.7 years and a mean disease duration of 14 years, had high disease activity as shown by BASDAI (mean: 6.1) and ASDAS (mean: 3.7) and in all patients risk factors for radiographic spinal progression were present with an elevated CRP in 69.7% or of at least one syndesmophyte at baseline in 50.5%. Prior treatment with bDMARDs was recorded in 31.5% in the combination therapy group vs 16.4% in the monotherapy arm, and the mean duration of prior biologic therapy was 2.3 years (95% CI: 1.42 to 3.13) and 5.11 years (95% CI: 2.04 to 8.18) in these groups, respectively (p=0.13).

**Table 2:** Baseline characteristics of randomised patients in the CONSUL study

Parameters		Combination therapy (GOL+CEL) N=54		Monotherapy (GOL alone) N=55		All randomised patients N=109	
		Valid, n	Value	Valid, n	Value	Valid, n	Value
Sex, male	n (%)	54	40 (74.1)	55	41 (74.5)	109	81 (74.3)
Age, years	Mean (SD)	54	39.9 (9.9)	55	37.5 (10.8)	109	38.7 (10.4)
Disease duration, years	Mean (SD)	51	14.2 (10.3)	54	13.8 (10.1)	105	14 (10.1)
HLA-B27 positivity	n (%)	54	45 (83.3)	51	47 (92.2)	105	92 (87.6)
Current smoking	n (%)	53	19 (35.8)	55	22 (40)	108	41 (38)
Prior bDMARDs	n (%)	54	17 (31.5)	55	9 (16.4)	109	26 (23.9)
BASDAI	Mean (SD)	54	6.2 (1)	55	6.1 (1.1)	109	6.1 (1.1)
BASFI	Mean (SD)	54	5.7 (1.5)	55	4.7 (1.9)	109	5.2 (1.8)
ASAS-HI	Mean (SD)	54	8.4 (2.8)	55	7.2 (3.2)	109	7.8 (3.1)
BASMI	Mean (SD)	54	2.6 (1.9)	54	2.9 (1.4)	108	2.8 (1.6)
CRP, mg/L	Median (IQR)	54	9.2 (4.1; 18.5)	55	9.2 (3.2 ;27)	109	9.2 (3.7 ;20)
Elevated CRP (>5 mg/L)	n (%)	54	38 (70.4)	55	38 (69.1)	109	76 (69.7)
ASDAS	Mean (SD)	54	3.6 (0.6)	55	3.7 (0.9)	109	3.7 (0.8)
Presence of ≥1 syndesmophyte(s)	n (%)	54	27 (50)	55	28 (50.9)	109	55 (50.5)
mSASSS	Mean (SD)	54	13.5 (16.9)	55	10.3 (13.2)	109	11.9 (15.2)

ASAS-HI, Assessment of Spondyloarthritis International Society Health Index; ASDAS, Axial Spondyloarthritis Disease Activity Index; BASDAI, Bath Ankylosing Spondylitis Disease Activity Index; BASFI, Bath Ankylosing Spondylitis Functional Index; BASMI, Bath Ankylosing Spondylitis Metrology Index; bDMARD, biological disease modifying anti-rheumatic drug; CEL, celecoxib; CRP, C reactive protein; GOL, golimumab; HLA-B27, human leucocyte antigen; mSASSS, Modified Stoke Ankylosing Spondylitis Spinal Score.

All patients in the combination therapy group who completed the study per protocol received celecoxib. None of the patients of the combination therapy group received other NSAIDs after week 12. In the golimumab monotherapy arm, 23 (42.6%) patients received NSAIDs at least once in the core phase. The mean (SD) ASAS NSAID intake score was 96.40 (7.51) in the combination therapy group and 5.53 (12.13) in the golimumab monotherapy group.

### Radiographic spinal progression

#### Main analysis

There were 12 patients (9 patients in the combination therapy group and 3 patients in the monotherapy group) with missing mSASSS scores at year 2, which were imputed for the primary analysis.

**Figure 3:**
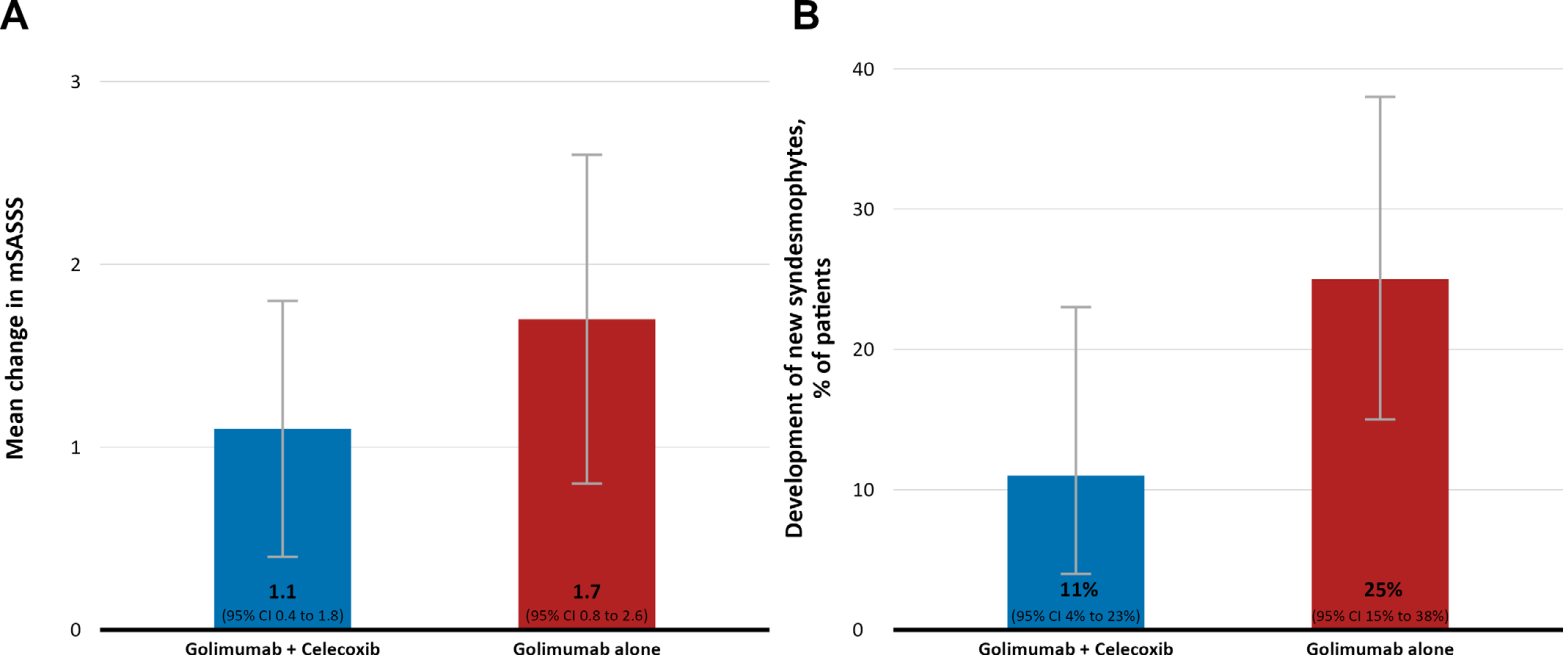
The mean mSASSS change (A) and the proportion of patients with new syndesmophytes (B) over 2 years of treatment in the intention-to-treat population of the COmparison of the effect of treatment with NSAIDs added to anti-TNF therapy versus anti-TNF therapy alone on progression of structural damage in the spine over two years in patients with ankyLosing spondylitis study. mSASSS, modified stoke ankylosing spondylitis spinal score.

**Figure 4:**
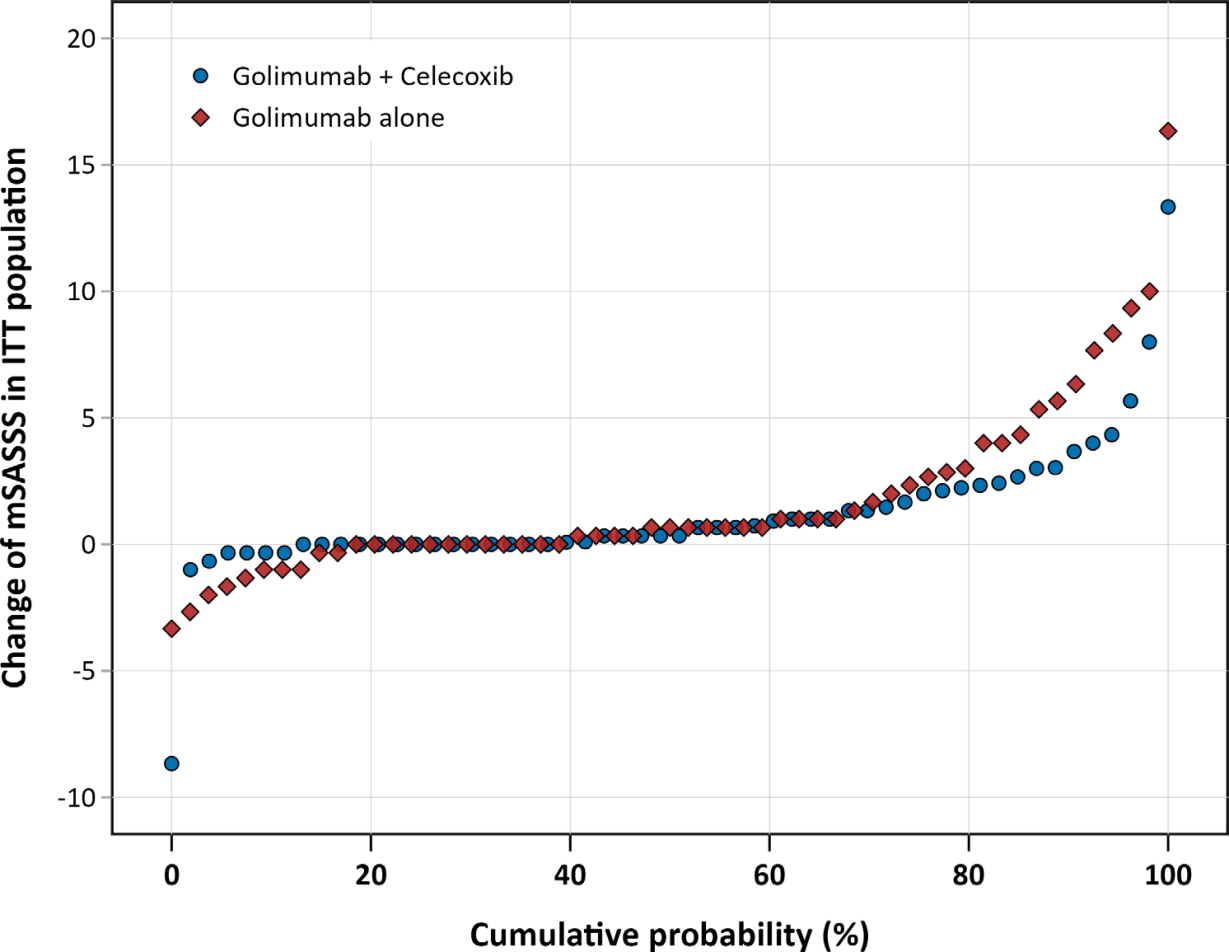
Cumulative probability plot of radiographic progression in the COmparison of the effect of treatment with NSAIDs added to anti-TNF therapy versus anti-TNF therapy alone on progression of structural damage in the spine over two years in patients with ankyLosing spondylitis study. ITT, intention to treat; mSASSS, modified stoke ankylosing spondylitis spinal score.

The mean mSASSS change over 2 years of treatment in the ITT analysis was numerically higher in the monotherapy group (1.7; 95% CI 0.8 to 2.6) when compared with the combination therapy group (1.1; 95% CI 0.4 to 1.8) (figure 3A), although this difference did not reach statistical significance (p=0.79). On an individual patient level, the mSASSS progression over 2 years is depicted in a cumulative probability plot (figure 4). There was no statistically significant difference in the proportion of patients who developed new syndesmophytes (25% vs 11% in the monotherapy group versus the combination therapy group, p=0.12—figure 3B) nor in the proportion of patients with new syndesmophytes or with the growth of existing syndesmophytes (27% vs 11% in the monotherapy group versus the combination therapy group, p=0.07).

#### Sensitivity analyses

In the longitudinal analysis using a linear mixed effects model adjusted for baseline mSASSS, we found no significant difference between the combination and monotherapy groups in terms of mSASSS change over time. The estimate for the contrast between the two groups (monotherapy vs combination therapy) was 0.43 (95% CI −1.49 to 2.34), further confirming the absence of a significant treatment effect among the groups (table 3). In the ANCOVA analysis adjusted for baseline mSASSS and the time from baseline radiography to screening, the least square means of the mSASSS progression were similar to that of the primary analysis: 1.8 (95% CI 0.9 to 2.6) and 1.0 (95% CI 0.2 to 1.9) for the monotherapy group and combination therapy group, respectively. The same was true for the model adjusted for the baseline mSASSS and the history of bDMARDs treatment: 1.7 (95% CI 0.9 to 2.5) and 1.1 (95% CI 0.2 to 1.9) for the monotherapy group and the combination therapy group, respectively.

**Table 3:** Longitudinal linear mixed-effect models to evaluate the time course of mSASSS between groups

Parameter	Estimate	95% CI	P value
Treatment groups (monotherapy vs combination)	0.43	−1.49 to 2.34	0.66
Baseline mSASSS	1.03	1.01 to 1.05	<0.001
Time point (year 2 vs BSL)	1.63	0.78 to 2.48	<0.001
Time point×treatment groups	−0.51	−1.74 to 0.72	0.41

BSL, baseline; mSASSS, Modified Stoke Ankylosing Spondylitis Spinal Score.

In total, 97 patients (monotherapy group: n=52 and combination therapy group: n=45) had complete mSASSS scores with ≥20 vertebral edges scored at baseline and at year 2. These patients were included in the completer analysis. Also in this analysis, there was no significant difference between the groups: the mean mSASSS progression changes were 1.6 (95% CI 0.7 to 2.6) and 1.1 (95% CI 0.1 to 2.1) for the monotherapy arm and combination therapy group, respectively.

After exclusion of three patients from the combination therapy group with an NSAID intake index of <80 and of six patients from the monotherapy group with an NSAID intake index of >20 in the core phase of the study did not change the results substantially: 1.8 (95% CI 0.8 to 2.9) vs 1.4 (95% CI 0.7 to 2.0) mSASSS points in the monotherapy group versus the combination therapy group, respectively.

We also compared the change in mSASSS in subpopulations of patients with different risks of progression at baseline. In 65 patients with elevated CRP (>5 mg/L) at baseline, the mean change in mSASSS was 1.9 (95% CI 0.6 to 3.2) in the golimumab monotherapy group (n=35) and 1.1 (95% CI 0.4 to 1.8) in the combination therapy group (n=30)—online supplemental file 3. In 54 patients with syndesmophyte(s) at baseline, the mean change in mSASSS was 2.7 (95% CI 1.0 to 4.4) in the golimumab monotherapy group (n=29) and 1.8 (95% CI 0.2 to 3.4) in the combination therapy group (n=25)—online supplemental file 3. Finally, we assessed 30 patients with both elevated CRP and syndesmophyte(s) at baseline: the mean change in mSASSS was 3.4 (95% CI 1.0 to 5.9) in the golimumab monotherapy group (n=18) and 2.4 (95% CI 0.8 to 4.1) in the combination therapy group (n=12)—online supplemental file 3. The ICC for the assessment of the spinal radiographs among the three readers was excellent for the status scores (0.94 for baseline and 0.95 at year 2) and moderate for the change score (0.67).

10.1136/ard-2023-224699.supp3 Supplementary data

### Clinical outcomes

After the initial improvement observed in all randomised patients during the run-in phase of the study, all investigated clinical outcome measures remained stable during the core phase of the study without major differences between the treatment groups—online supplemental file 3. We performed multivariable linear regression analyses adjusting for the baseline covariates that are potentially associated with the outcome, and did not observe any significant difference in the change in disease activity (BASDAI and ASDAS) between the monotherapy and combination therapy groups (online supplemental file 3).

### Safety assessments

The treatment groups did not show significant differences in terms of AEs, AEs of special interest and SAEs. Throughout the study, a total of 702 AEs occurring in 111 patients were reported (327 in the monotherapy group; 353 AEs in the combination therapy group; and 22 before randomisation). AEs of special interest included 318 infections (150 in the monotherapy group and 6 before randomisation; 162 in the combination therapy group) and 3 major cardiovascular events (2 events in the monotherapy group—1 pulmonary embolism and 1 thrombophlebitis and one deep vein thrombosis in the combination therapy group). Moreover, 2 cases of creatinine increase were documented in the combination therapy group and 24 events of liver enzyme elevations (11 in the combination therapy group, 12 in the monotherapy group and 1 before randomisation) were observed. A total of 14 SAEs were reported until week 112 (6 in the combination therapy group, 5 in the monotherapy group and 3 before randomisation), with no differences between both treatment groups (table 4). Study discontinuation due to AEs was numerically higher in the combination therapy group (n=4; dry cough; deep vein thrombosis; dyspnoea; chronic focal encephalitis) compared with the monotherapy arm (n=1; pulmonary embolism).

**Table 4:** Serious adverse events observed in the core phase of the CONSUL study

SAE categories	GOL+CEL Exposure: 92.9 PY		GOL alone Exposure: 104.5 PY	
	N	EAIR, per 100 PY	N	EAIR, per 100 PY
Infections and infestations (total)	1	1.1	2	1.91
Skin infection	–	–	1	0.96
Tendon infection	–	–	1	0.96
Acute gastroenteritis	1	1.1	0	0
Psychiatric disorders (total)	1	1.1	0	0
Worsening of known depression	1	1.1	–	–
Nervous system disorders (total)	1	1.1	0	0
Chronic focal encephalitis	1	1.1	–	–
Cardiovascular disorders (total)	0	0	1	0.96
Pulmonary embolism	–	–	1	0.96
Gastrointestinal disorders (total)	2	2.2	1	0.96
Appendicitis	1	1.1	–	–
Gastritis	–	–	1	0.96
Haematochezia	1	1.1	–	–
Musculoskeletal disorders (total)	1	1.1	1	0.96
Meniscus injury	1	1.1	1	0.96

CEL, celecoxib; EAIR, exposure adjusted incidence rate (per 100 patient-years); GOL, golimumab; PY, patient-year; SAE, serious adverse event.

## Discussion

In this randomised-controlled study, we did not find a significant difference between the treatment arms, although the addition of celecoxib to golimumab monotherapy resulted in numerically less radiographic spinal progression.

We compared two treatment strategies in terms of their ability to retard radiographic spinal progression in patients with r-axSpA—monotherapy with the TNFi golimumab versus combination of golimumab with the selective COX-II inhibitor celecoxib. Celecoxib has demonstrated evidence for retardation of radiographic spinal progression when given continuously over 2 years, possibly attributed to its direct inhibitory effect on osteoblast activity. Golimumab is a representative of a drug class with a delayed effect on radiographic spinal progression, which may be explained by its direct inhibitory effect on new bone formation and indirect inhibitory effect via inhibition of inflammation. We hypothesised that adding celecoxib to treatment with the TNFi golimumab would be associated with an improved structural outcome (as reflected by the change in mSASSS) as compared with TNFi therapy alone in r-axSpA who are at high risk for progression.

In the primary analysis, we observed a numerical difference in the mSASSS progression over 2 years between the treatment arms (1.7 for the golimumab monotherapy and 1.1 for the combined therapy with golimumab and celecoxib) that did not achieve statistical significance but also was clearly below the anticipated 1.5-point difference that deemed clinically relevant. All secondary analyses including analyses accounting for baseline structural damage, elevated CRP, previous use of bDMARDs, NSAID intake during the study, analysis of completers and analysis focusing on syndesmophyte formation/progression, were in line with the primary analysis.

What is the practical relevance of these findings? According to the current management recommendations for axSpA, NSAIDs are considered as a treatment option for symptomatic patients with axSpA and can be given also in combination with bDMARDs (including TNFi) if this is necessary for a better control of disease activity. In patients with a good control of disease activity with a bDMARDs, no NSAIDs are required.^28^ This strategy is reinforced now by the results of the CONSUL study: even in patients with high risk of structural damage progression (patients with syndesmophytes and/or elevated CRP), NSAIDs should be given in addition to bDMARDs only if clinically indicated. At the same time, our results showing a numerical difference in the mSASSS progression and syndesmophyte progression might help to make a decision to use an NSAID (COX-II inhibitors) more consequently in patients, who are not symptom free and inflammation free despite use of TNFi.

We did not observe major differences on the group level in terms of clinical outcomes, meaning that a sufficient disease activity control is well possible with TNFi alone, and NSAIDs should only be given if this is not the fact. The tolerability of both treatment strategies was comparable; however, we observed more dropouts in the combined treatment arm.

The main strength of the study is the randomised and controlled study design that allowed for a valid comparison of the strategies. Preselection of good responders to TNFi in the run-in phase allowed for a homogeneous study group and for a relatively low dropout rate in both arms. Finally, selection of patients at high risk of radiographic progression allowed for detection of progression in the study and of the treatment effect.

Limitations of the study include the relatively small number of patients per group. Although this power calculation for our study was based on the assumptions of previously published historical data, it is evident that future studies will require a significantly larger sample size to reliably detect the differences observed between the two treatment groups based on the presented results. The study results are valid for celecoxib and possibly for other COX-II inhibitors, but it is unknown whether the same data would have been observed with a non-selective COX inhibitor such as diclofenac, which does not appear to have an effect based on previous studies.^15^ It should also be noted that NSAID use was also allowed in the monotherapy group. However, we consider it unlikely that a major effect was missed because the difference in the ASAS NSAID score was large (96.40 in the combination therapy group and 5.53 in the golimumab monotherapy group). For comparison, in the previous celecoxib trial,^13^ the difference in total drug dose was small (mean daily celecoxib dose was 243 mg in the continuous group and 201 mg in the on-demand group), and not all patients remained on celecoxib. On another note, the results are likely true for the entire group of TNFi, but may not be generalisable to other bDMARDs (ie, interleukin 17 inhibitors) and targeted synthetic DMARDs (ie, Janus kinase inhibitors). In addition, it should be emphasised that the treatment was not blinded during the core phase of the study. To overcome such a potential bias on the primary endpoint, the scoring of the spinal radiographs was performed blinded for treatment groups (and also time point of the assessment). Furthermore, it needs to be noted, that there was an imbalance in terms of prior use of bDMARDs, with a higher proportion of bDMARD-experienced patients in the combination therapy group, which may have affected the results. Finally, the study cannot answer the question if there would have been a difference between the strategies after a longer observation period, for example, after 4 years.

In conclusion, the combination of celecoxib with golimumab did not show significant superiority over golimumab monotherapy in retarding radiographic spinal progression over 2 years in r-axSpA patients. The observed numerical difference in favour of combined treatment might be, however, relevant for patients at high risk in a situation, where combination therapy is indicated clinically.

## Data Availability

Data are available upon reasonable request. Deidentified participant data can be made available after approval of a written request for scientific purposes by the corresponding author.
