# Supplementary material for: Comparison of the effect of treatment with NSAIDs added to anti-TNF therapy versus anti-TNF therapy alone on the progression of structural damage in the spine over 2 years in patients with radiographic axial spondyloarthritis from the randomised-controlled CONSUL trial
Source: Ann Rheum Dis. 2024 Jan 16;83(5):599–607. doi: 10.1136/ard-2023-224699 (PMC11041582; doi:10.1136/ard-2023-224699)
Supplement: Supplementary data [file ard-2023-224699supp002.pdf]

BMJ Publishing Group Limited (BMJ) disclaims all liability and responsibility arising from any reliance placed on this supplemental material which has been supplied by the author(s)

Treatment assignment

Subjects who responded to treatment with golimumab at week 12 were randomized 1:1 by an FDA 21 part 11 compliant software tool (SecuTrial) of the Coordinating Center for Clinical Studies of the Charité Berlin (KKS Koordinierungszentrum für Klinische Studien) in order to receive golimumab plus continuous treatment with celecoxib vs. golimumab alone.

In the frist step **block randomization** was applied stratified by study centre and the time period between the first radiographic examination used as “baseline radiographic examination” and screening visit. Three months intervals (0-3, 4-6 etc. months) were considered to build up these strata. However, this additional stratification by study centre, which could not be excluded since it is a necessary part of the SecuTrial software, caused an imbalance between the treatment groups within the strata defined by the time lag between “baseline radiographic examination” and screening.

To solve this problem the randomization method was changed on April 16th 2018 and 40 patients were then randomized to the alternative path as described below:

Firstly, only the time lag intervals were used for the definition of strata but not the study centre in addition. Secondly, to achieve again a balance between the treatment groups **a biased coin algorithm** was applied which takes the randomization history into account and aims balance between the treatment groups within the strata. For this reason, Atkinsons’s DA-optimal biased coin algorithm (1) was used. In this second step strata specific random lists were generated at the German-Rheumatism-Research Center (DRFZ) by a statistician not otherwise involved in the CONSUL trial. According to these random lists an eligible patient was assigned to a treatment group by using the next random code within the stratum she/he belongs to.

1. *Atkinson AC. Optimum biased-coin designs for sequential treatment allocation with covariate information. Stat Med. 1999 Jul 30;18(14):1741-52; discussion 1753-5. doi: 10.1002/(sici)1097-0258(19990730)18:14<1741::aid-sim210>3.0.co;2-f. PMID: 10407244.*
