# Supplementary material for: Comparison of the effect of treatment with NSAIDs added to anti-TNF therapy versus anti-TNF therapy alone on the progression of structural damage in the spine over 2 years in patients with radiographic axial spondyloarthritis from the randomised-controlled CONSUL trial
Source: Ann Rheum Dis. 2024 Jan 16;83(5):599–607. doi: 10.1136/ard-2023-224699 (PMC11041582; doi:10.1136/ard-2023-224699)
Supplement: Supplementary data [file ard-2023-224699supp001.pdf]

CLINICAL STUDY PROTOCOL

COmparison of the effect of treatment with **N**SAIDs added to anti-TNF therapy versus anti-TNF therapy alone on progression of **S**tr**U**ctural damage in the spine over two years in patients with anky**L**osing spondylitis: a randomized controlled multicentre trial (**CONSUL**)

Protocol number: CONSUL2016
Protocol version: 1.3 including Amendment No. 1
Date: 13.02.2017
EudraCT Number: 2016-000615-33

Investigational Products: Golimumab, Celecoxib
Study Phase: phase IV
Study Design: randomized, controlled, prospective, open-label, multicentrestudy

Sponsor: Charité Universitätsmedizin Berlin
Charitéplatz 1
10117 Berlin, Germany

Coordinating Investigator: Prof. Dr. med. Denis Poddubnyy
Medizinische Klinik für Gastroenterologie,
Infektiologie und Rheumatologie
Campus Benjamin Franklin
Charité Universitätsmedizin Berlin
Hindenburgdamm 30
12203 Berlin, Germany
Tel: +49 30 8445-2302 or 450-514544
Fax: +49 30 8445-4149
Email: denis.poddubnyy@charite.de
Study Physician: Dr. med. Burkhard Muche
-same address-
Tel. +49 (30) 8445-4144
Email: burkhard.muche@charite.de

Proft F, *et al. Ann Rheum Dis* 2024; 83:599–607. doi: 10.1136/ard-2023-224699

TABLE OF CONTENT

1 LIST OF ABBREVIATIONS 4

2 PROTOCOL SYNOPSIS 6

3 ASSESSMENT SCHEDULE 13

4 INTRODUCTION 15

4.1 Prevalence and Clinical Manifestations of Ankylosing Spondylitis 15
4.2 Current Treatment of Ankylosing Spondylitis 15
4.3 Evidence for disease modification in Ankylosing Spondylitis 16
4.3.1 NSAIDs 16
4.3.2 TNF blocker 16
4.3.3 Combination of NSAIDs and TNF blocker 16

5 STUDY OBJECTIVE 18

5.1 Primary Endpoint 18
5.2 Secondary Endpoints 18

6 INVESTIGATIONAL PLAN 20

6.1 Study Design 20
6.2 Justification of Design aspects 21
6.2.1 Control(s) / Comparator(s) 21
6.2.2 Dose, Mode, Duration and Scheme of Intervention 22
6.3 Study Population 23
6.3.1 Inclusion Criteria 23
6.3.2 Exclusion Criteria 24
6.4 Prior and Concomitant Therapy; Prohibited Medications 26
6.5 Study Treatment 27
6.5.1 Treatments Administered 27
6.5.2 Rescue Treatments 27
6.5.3 Drug supplies, Packaging and Labelling 28
6.5.4 Storage, Disposition and Dug Accountability 29
6.5.5 Treatment Compliance 29
6.6 Study Discontinuation 30
6.6.1 Discontinuation of Individual Subjects 30
6.6.2 Discontinuation of the Entire Study 31
6.7 Study Procedures 31

7 SAFETY CONSIDERATIONS 36

7.1 Definition and Reporting of Adverse Events 36
7.2 Definition and Reporting of a Serious Adverse Event 37
7.2.1 Definition of a Serious AE 37
7.2.2 Reporting a Serious AE 38
7.3 Pregnancy 39
7.4 Data and Safety Monitoring Committee (DSMC) 39

8 PLANNED TREATMENT OF THE PATIENTS AFTER STUDY END 39

9 OVERALL RISK/BENEFIT ASSESSMENT 41

9.1 Celecoxib 41

Proft F, *et al. Ann Rheum Dis* 2024; 83:599–607. doi: 10.1136/ard-2023-224699

9.2 Golimumab 41
10 ETHICAL ASPECTS 43
10.1 Declaration of Helsinki / Good Clinical Practise (GCP) 43
10.2 Patient Information and Informed Consent 43
10.3 Insurance coverage 44
11 INDEPENDENT ETHICS COMMITTEE AND REGULATORY AUTHORITIES 45
11.1 Approval of the Study by Regulatory Authorities and Independent Ethics Committees45
11.2 Notification of the Study 45
11.3 Report and Documentation Obligation 45
12 CONDITIONS FOR MODIFYING THE PROTOCOL 46
13 STATISTICAL ANALYSIS PLAN 47
13.1 Definition of Population for Analysis 47
13.2 Intent-to-Treat Population 47
13.3 Per Protocol Population 47
13.4 Safety Population 47
13.5 Efficacy analyses 48
13.6 Safety Data Analysis 48
13.7 Methods against Bias 49
13.8 Sample Size and Power Calculation 49
14 QUALITY CONTROL AND QUALITY ASSURANCE / MONITORING 51
14.1 Monitoring 51
14.2 Study Documentation, CRFs and Record Keeping 51
14.3 Investigator's Files/Retention of Documents 52
14.4 Source Documents and Background Data 53
14.5 Audits and Inspections 54
14.6 Case Report Forms 54
15 CONFIDENTIALITY OF TRIAL DOCUMENTS AND SUBJECT RECORDS 55
16 REFERENCES 56
17 SIGNATURE PAGE 61

Proft F, *et al. Ann Rheum Dis* 2024; 83:599–607. doi: 10.1136/ard-2023-224699

1 LIST OF ABBREVIATIONS

AE – Adverse Event

ALT - Alanine amino-Transferase

AMG – German Medicinal Product Act (The Drug Law)

AP - Alkaline Phosphatase

AS – Ankylosing Spondylitis

ASDAS - Ankylosing Spondylitis Disease Activity Score

ASAS HI - Ankylosing Spondylitis Health Index

AST - Aspartate amino-Transferase

BASDAI - Bath Ankylosing Spondylitis Disease Activity Index

BASDAI50 – improvement of 50% or more from the baseline BASDAI score

BASFI - Bath AS Functional Index

BASMI - Bath AS Metrology Index

CRP - C-reactive Protein

COX-II – Cyclooxygenase-II

CRF – Case Report Form

DMARDs - Disease-Modifying Anti-Rheumatic Drugs

ESR – Erythrocyte Sedimentation Rate

ET – Early Termination of the study

EU – European Union

GCP – Good Clinical Practice

GESPIC – GErman SPondyloarthritis Inception Cohort

GGT - Gamma-Glutamyl-Transferase

HBV – Hepatitis B Virus

ICH – International Conference of Harmonisation

IEC - Independent Ethics Committee

IGRA – Interferon Gamma Release Assay (serum test for tuberculosis)

IL - Interleukin

IRB – Institutional Review Board

ITT – Intention-To-Treat

MHC – Major Histocompatibility Complex

MRI - Magnetic Resonance Imaging

mSASSS - modified Stoke Ankylosing Spondylitis Spine Score

NRS – Numeric Rating Scale

NSAID - Non-Steroidal Anti-Inflammatory Drug

PASS – Patient Acceptable Symptom State

PhASS - Physician Acceptable Symptom State

PPI – Proton pump Inhibitor

SC – subcutaneously

SpA - Spondyloarthritis

SUSAR - Suspected Unexpected Serious Adverse Reaction

TB - Tuberculosis

TNF - tumour necrosis factor

Proft F, *et al. Ann Rheum Dis* 2024; 83:599–607. doi: 10.1136/ard-2023-224699

2 PROTOCOL SYNOPSIS

**Protocol title:**	Comparison of the effect of treatment with NSAIDs added to anti-TNF therapy versus anti-TNF therapy alone on progression of structural damage in the spine over two years in patients with ankylosing spondylitis: a randomized controlled multicentre trial (CONSUL)
**Protocol number**	CONSUL2016
**Protocol version:**	1.3 including Amendment No. 1
**Version date:**	13.02.2017
**Sponsor:**	Charité Universitätsmedizin Berlin<br>Charitéplatz 1<br>10117 Berlin, Germany
**Coordinating investigator:**	Prof. Dr. Denis Poddubnyy<br>Department of Rheumatology,<br>Campus Benjamin Franklin<br>Charité Universitätsmedizin Berlin<br>Hindenburgdamm 30<br>12203 Berlin, Germany
**Study sites:**	21
**Study phase:**	IV
**Study design:**	Randomized, controlled, open-label multicenter study
**Indication:**	Ankylosing spondylitis (AS)
**Study population:**	Patients with active AS who have had an inadequate response to NSAIDs
**Number of subjects:**	170
**Investigational products:**<br><br>**Reference Therapy:**	Golimumab 50 mg SC every 4 weeks (pre-filled syringe) and<br>Celecoxib 400 mg per os daily (capsules)<br><br>Golimumab 50 mg SC every 4 weeks (pre-filled syringe) alone
**Duration of the study:**	112 weeks (108 weeks of therapy + 4 weeks of follow-up for safety)
**Study objective:**	To evaluate the impact of treatment with a non-steroidal anti-inflammatory drug (NSAID) – Celecoxib – when added to anti-tumour necrosis factor (TNF) therapy – Golimumab – as compared to anti-TNF therapy (Golimumab) alone on progression of structural damage in the spine over two years in patients with AS.
**Study design and methodology:**	This study is a randomized, controlled multicentre clinical trial. The study consists of a 6-week screening period, a 12-week period (Phase I: Run-in Phase) of treatment with Golimumab for all subjects followed by a 96-week controlled treatment period (Phase II: Core-Phase) with Golimumab plus Celecoxib versus Golimumab alone, and a safety follow-up period of 4 weeks. Only subjects with a good clinical response to Golimumab in the Phase I (achievement of ≥2 absolute points on the BASDAI 0-10 scale response at Week 12) will be eligible for Phase II and will be randomized 1:1 ratio to receive Golimumab plus Celecoxib or Golimumab alone for 96 weeks. At week 108 the primary study endpoint (radiographic spinal

Proft F, *et al. Ann Rheum Dis* 2024; 83:599–607. doi: 10.1136/ard-2023-224699

	progression as assessed by the modified ankylosing spondylitis spine score – mSASSS – change) will be evaluated.
**Selection criteria:**	***Inclusion Criteria***<br>*Phase I of the study:*<br>1. Age ≥18 years.<br>2. Definite diagnosis of AS according to the modified New York criteria[1].<br>3. History of an inadequate response to adequate therapeutic trials with NSAID´s defined as at least two NSAID´s over a 4-week period in total at maximum recommended or tolerated anti-inflammatory dose<br>4. Active disease as defined by a BASDAI value of ≥4.0 at screening.<br>5. Presence of at least one of the following risk factors for radiographic spinal progression:<br>a. Elevated CRP (>5mg/l) at screening and the absence of reasons for elevated CRP other than AS;<br>b. Presence of ≥ 1 syndesmophyte on prior X-rays of the spine.<br>6. Subject is a candidate for anti-TNF therapy based on the investigator's opinion.<br>7. Subject is able and willing to give a written informed consent and comply with the requirements of the study protocol. Only patients who gave written informed consent will be included in the trial.<br>8. <u>If female</u>: either unable to bear children (postmenopausal for at least 1 year or surgically sterile) or is willing and able to practice a reliable method of contraception throughout the study and 6 months after the last dose of study drug. A highly effective method of birth control is defined as those which result in a low failure rate (i.e. less than 1% per year) when used consistently and correctly such as implants, injectables, combined oral contraceptives or a vasectomized partner (at least 1 year prior to enrolment). In questionable cases of menopausal status a blood sample with simultaneous levels of follicle stimulating hormone (FSH) above 40 U/l and estradiol below 30 nl/l is confirmatory. The results of the pregnancy test performed at Screening must be negative.<br>9. If on NSAIDs: the dose must be stable for at least 2 weeks prior to baseline.<br>10. If on analgesics: the dose must be stable for at least 2 weeks prior to baseline.<br><br>*Phase II of the study*:<br>1. Achievement of ≥2 absolute points (on a 0-10 scale) reduction of the BASDAI after 12 weeks of Golimumab treatment in the Phase I.<br><br>***Exclusion Criteria***<br>1. For female subjects: pregnancy or lactating.<br>*2.* Subjects with chronic inflammatory articular disease other than SpA or systemic autoimmune

		diseases, e.g. systemic lupus erythematosus, Sjögren´s syndrome, rheumatoid arthritis. *Other manifestations of SpA such as psoriasis, uveitis and peripheral arthritis are not regarded as exclusion criteria.*<br>3. History of inadequate response (primary non-response) to previous anti-TNF therapy. Patients who discontinued previous anti-TNF therapy or other biologics for reasons other than primary non-response may participate in the study if anti-TNF therapy is considered to be suitable in the opinion of the investigator. This prior therapy must have been terminated at least 3 months (in case of TNF blockers or secukinumab) or 5 half-lifes in the case of other biologics prior to Baseline visit.<br>4. Treatment with any other investigational drug within 3 months of 5 half-lifes of the drug (whichever is longer) prior to Baseline visit.<br>5. History of intolerability or hypersensitivity reaction to Golimumab.<br>6. History of intolerability or hypersensitivity reaction to Celecoxib or sulphonamides.<br>7. Previous reactions of asthma, urticaria or other severe hypersensitivity/reactions after intake of acetylsalicylic acid or other NSAIDs.<br>8. Known hypersensitivity to any component of the study medications.<br>9. Treatments with DMARDs other than methotrexate or sulfasalazine within 4 weeks prior to baseline (in case of leflunomide either 8 weeks or 4 weeks with a standard cholestyramine wash-out).<br>10. The presence of total spinal ankylosis.<br>11. Treatment with intravenous, intramuscular or intraarticular/periarticular steroids within 4 weeks prior to Baseline Visit; treatment with oral steroids in a dose of >10 mg (prednisolone equivalent) per day.<br>12. Any active current infection. History of recurrent clinically significant infection (suggestive for primary or secondary immunodeficiency). Infections requiring treatment with antibiotics within 4 weeks prior to baseline.<br>13. Current clinical signs and symptoms suggestive for tuberculosis.<br>*14.* Positive tuberculosis serum IGRA test and/or abnormal chest x-ray (both performed at screening or within 3 months prior to screening) suggestive for past or present tuberculosis (positive x-ray). *Patients with a positive tuberculosis serum test but negative chest x-ray and without clinical symptoms suggestive for tuberculosis may participate in the study after initiation of standard prophylactic anti-tuberculous treatment according to local guidelines.*<br>15. Chronic infection with hepatitis B.<br>16. Actual malignancies or history of malignancies with curative treatment within 5 years prior to

		screening, except successfully treated non-metastatic squamous-cell or basal-cell carcinoma of the cutis or carcinoma in situ of the cervix.<br>17. History of demyelinating disease (like multiple sclerosis; including myelitis) or neurologic symptoms suggestive of demyelinating disease.<br>18. History of cardiovascular events: myocardial infarction, stroke or transient ischemic attack, pulmonary embolism.<br>19. Diagnosis of the coronary heart disease or peripheral arterial occlusive disease; uncontrolled arterial hypertension.<br>20. High cardiovascular risk as assessed by the presence of >2 cardiovascular risk factors (e.g., uncontrolled hypercholesterolemia, diabetes mellitus, smoking, uncontrolled arterial hypertension).<br>21. History of oesophageal, gastric, pyloric channel or duodenal ulceration documented by endoscopy/radiographic examination at any time before the screening visit, or any clinically relevant gastrointestinal bleeding, perforation, or gastric outlet obstruction.<br>22. History of a chronic inflammatory bowel disease (Crohn's disease or ulcerative colitis).<br>23. Evidence of other severe uncontrolled gastrointestinal, hepatic (serum albumin <25 g/l or Child-Pugh-Score >10), renal, pulmonary, cardiovascular, nervous or endocrine disorders.<br>24. Increased bleeding risk including therapy with anticoagulants and thrombocyte aggregation inhibitors (e.g. low-dose aspirin).<br>25. Chronic or acute congestive heart failure (NYHA II to IV).<br>26. Patients with a history of a severe psychiatric illness, which might interfere with the patient's ability to understand the requirements of the study and assessment.<br>27. Diagnosis of fibromyalgia.<br>28. Alcohol abuse or illegal drug consumption in the last 12 months.<br>29. Vaccination with a live vaccine within 12 weeks prior to baseline.<br>30. Any of the following laboratory abnormalities as detected at screening:<br>Haemoglobin < 8.5 g/dl<br>Neutrophil counts < 2.000 / µl<br>Platelet count < 125.000 / µl<br>Aspartate aminotransferase (AST) or alanine aminotransferase (ALT) or alkaline phosphatase (AP) or gamma-glutamyl-transpeptidase (GGT) >3 times upper limit of normal<br>Creatinine > 1.4 mg/dl (= 120 µmol/l)<br>31. Patients who are defined as vulnerable patients according to Art. 10 REGULATION (EU) No 536/2014: patients who are institutionalised due to regulatory or juridical order; patients who are an

	employee of the investigator or study site, with direct involvement in the proposed study or other studies under the direction of that investigator or study site; family members of the employees or the investigator.<br>32. Any other conditions making the patient unsuitable in the opinion of the investigator for the participation in the current study.<br>33. For patients participating in the MRI sub-study only: patients with contraindications for the magnetic resonance imaging (MRI) including but not limited to: claustrophobia, seizure disorders, presence of a implanted electronic devices (e.g., heart pacemaker, insulin pump, etc.), metal implants and metal foreign bodies in the patient's body suspected to be ferromagnetic, tattoos performed with metal-containing paints or tattoos of the large skin areas.
**Criteria for evaluation:**	**Efficacy:**<br>*Primary endpoint:*<br>• Radiographic spinal progression measured by the change in the modified Stoke Ankylosing Spondylitis Spine Score (mSASSS) over two years of therapy.<br><br>*Secondary endpoints:*<br>• New syndesmophyte formation or progression of existing syndesmophytes after 2 years of treatment.<br>• Improvement of disease activity, function, axial mobility and quality of life measures at week 12 and week 108 in comparison to baseline according to:<br>– BASDAI<br>– ASDAS<br>– CRP and erythrocyte sedimentation rate (ESR)<br>– Bath AS Functional Index (BASFI)<br>– Bath AS Metrology Index (BASMI) and chest expansion<br>– Global assessment (patient/physician), general pain and nocturnal pain on the numeric rating scale (NRS)<br>– ASAS Health Index<br>– Physician Acceptable Symptom State (PhASS)<br>– Patient Acceptable Symptom State (PASS)<br>– Percentage of subjects who achieve an ASAS20, ASAS40, ASAS partial remission, BASDAI50 and ASDAS in comparison to baseline;<br>• Change of the bone and cartilage biomarkers serum levels at week 108 in comparison to baseline and their relevance for the prediction of radiographic progression.<br>• Change of the enteric microbiome profile at week 108 in comparison to baseline.<br>• Change of osteitis score and scores for the chronic post-inflammatory changes in the spine and sacroiliac joints (SIJ) on magnetic resonance imaging (MRI) by Berlin MRI scoring method at week 108 in comparison to baseline – for the MRI sub-study only.<br><br>**Safety:**<br>Adverse events (AE), serious AE and AE of interest until Week 112.
**Statistical considerations:**	**Sample size:**<br>*To be assessed for eligibility:*

	n=190; assuming a 10% screening failure rate approximately.<br><br>*To be allocated to trial:*<br>n=170 patients with active AS despite treatment with NSAIDs alone will be included in the phase I of the trial and treated with Golimumab. Only patients with good clinical response after 12 weeks of treatment will be enrolled in the phase II of the trial. It is assumed that approximately 60% (n=100) of patients included in phase I will continue in the phase II of the study, considering that elevated CRP at baseline is currently the best response predictor for TNF-blocker therapy.<br><br>*To be analysed:*<br>n=100 (n=50 in each group) patients will enter phase II of the trial (intention-to-treat population - ITT) after 1:1 randomization.<br><br>**Statistical analysis:**<br>*Efficacy:*<br>Efficacy of the therapy in reduction of radiographic spinal progression will be assessed by the modified Stoke Ankylosing Spondylitis Spine Score (mSASSS)[2]. mSASSS is a radiographic score measuring structural changes in the lumbar and cervical spine. Images will be scored by 2 trained readers in a blinded manner that allows the open-label (although randomized) design of the trial.<br>The primary efficacy endpoint will be radiographic spinal progression assessed by the change in the mSASSS score over two years of therapy in both groups. The primary analysis will be based on the intention-to-treat population, a secondary one in patients with complete sets of radiographs at two time points. A two-sided Mann-Whitney U-test will be applied to compare the mSASSS progression between both treatment groups in both analyses.<br><br>*Safety:*<br>Safety outcome will include absolute number and percentage of patients who experienced adverse events (AE), serious AE, AE of interest (infections, malignancies, gastrointestinal events: ulceration, bleeding, perforation, gastric outlet obstruction; thrombotic cardiovascular events: myocardial infarction, stroke, pulmonary artery embolism, peripheral arterial of venous thrombosis; renal and hepatic function deterioration), as well as a proportion of patients who discontinued the study because of AEs.<br>In order to address possible long-term safety issues related to long term use of NSAIDs a safety Follow-Up Visit will be performed 3 months after discontinuation of the study treatment (either week 108 or the last study visit in the case of early discontinuation).<br>Safety population will include each patient who received at least one dose of Golimumab with or without Celecoxib. Statistical comparisons will be based on event rates per 100 patient years of exposure. Rates will be compared by exact Poisson methods.<br><br>*Secondary endpoint(s):*<br>Mann-Whitney U-test, linear mixed models, and Chi-square test will be used in order to evaluate differences in the secondary outcome parameters between treatment groups.
**Funding:**	German Federal Ministry of Education and Research (Bundesministerium für Bildung und Forschung), Grant No. FKZ 01KG1603<br><br>MSD Sharp & Dohme GmbH: providing Golimumab medication and funding for the MRI sub-study

Proft F, *et al. Ann Rheum Dis* 2024; 83:599–607. doi: 10.1136/ard-2023-224699

Proft F, *et al. Ann Rheum Dis* 2024; 83:599–607. doi: 10.1136/ard-2023-224699

3 ASSESSMENT SCHEDULE

**Study phase**	**Phase I**					**Phase II**									
	**Screening ≤ 6 weeks** (-42 days)	**Baseline (week 0)**	**Week 2*** (±7 days)	**Week 4*** (±7 days)	**Week 8*** (±7 days)	**Week 12*** (±7 days)	**Week 24*** (±14 days)	**Week 36*** (±14 days)	**Week 48*** (±14 days)	**Week 60*** (±14 days)	**Week 72*** (±14 days)	**Week 84*** (±14 days)	**Week 96*** (±14 days)	**Week 108*/ET[a]** (±14 days)	**Week 112 / FU[b]** (±14 days)
**Study Procedures**	Visit 1	Visit 2	Visit 3	Visit 4	Visit 5	Visit 6	Visit 7	Visit 8	Visit 9	Visit 10	Visit 11	Visit 12	Visit 13	Visit 14	Visit 15
Informed consent	X														
Inclusion/Exclusion criteria	X	X [c]				X									
Randomization						X									
Demographics	X														
Medical/Surgical history	X	X [c]													
Vaccination status	X														
Prior/concomitant medication	X	X	X	X	X	X	X	X	X	X	X	X	X	X	
Adverse events		X	X	X	X	X	X	X	X	X	X	X	X	X	X
Vital signs [d]	X	X	X	X	X	X	X	X	X	X	X	X	X	X	
Weight and height		X				X				X				X	
Smoking assessment [e]		X				X				X				X	
Physical examination	X	X	X	X	X	X	X	X	X	X	X	X	X	X	
Major AS assessment [f]		X				X		X		X		X		X	
Minor AS assessment [g]	X		X	X	X		X		X		X		X		
ECG [h]	X														
Blood analysis [i]	X [j]	X	X	X	X	X	X	X	X	X	X	X	X	X	
Biomarker blood samples [k]		X				X				X				X	
Urinalysis	X	X		X		X		X		X		X		X	
Serum pregnancy test [l]	X														
Stool samples [m]		X				X								X	
Hepatitis B screening (HBs-Ag, anti-HBc)	X														
Tuberculosis IGRA test [n]	X [n]														
Chest X-ray [n]	X [n]														
X-ray of the spine [o]	X [o]													X [o]	
MRI of spine and SIJ [p]	X [p]													X [p]	
Study drug – administration at study site		X		X	X	(X)									
Study drug issuance						X	X	X	X	X	X	X	X		
Returned drug account							X	X	X	X	X	X	X	X	

*Weeks after first Golimumab administration, study visits 3 to 6 have a time window of ±7 days, and the study visits 7 to 15 – a time window of ±14 days. If a subject has an out of window visit the next visit should occur according to the original schedule based on the date of the baseline visit (Visit 2 / Week 0)
a. ET = early termination visit only for subjects who prematurely discontinue study in the Phase II for any reason; should be performed within 4 weeks of last dose of study drug.
b. FU = Follow-up phone visit, collection of the safety information; to be performed 12 weeks after Week 108/ET visit.
c. Interim history.
d. Heart rate, blood pressure.
e. Former and actual smoking state at baseline, actual smoking status at subsequent visits.
f. Spinal mobility (BASMI), function (BASFI), disease activity (BASDAI, ASDAS, overall pain, night pain, PASS, PhASS), global assessment (patient/physician), overall health status / quality of life (ASAS health index).
g. BASDAI, ASDAS, PASS, PhASS, patient and physician global assessment only.
h. 12-lead electrocardiography (ECG).
i. Lab tests to be performed locally at the clinical trial site: Blood chemistry (total bilirubin, ALT, AST, GGT, AP, creatinine), hematology (erythrocytes, hemoglobin, leucocytes, neutrophils, lymphocytes, monocytes, basophils, eosinophils, platelets), CRP and ESR.
j. Lab test at screening should include also Quick-Test, International Normalized Ratio (INR) and activated Partial Thromboplastin Time (aPTT)
k. Biomarker blood samples (soluble and cellular markers of inflammation, cartilage and bone turnover) to be transferred to the Charité Universitätsmedizin Berlin.
l. Female patients of child-bearing potential only.
m. Stool samples for the microbiome analysis to be transferred to the Charité Universitätsmedizin Berlin.
n. Chest x-ray & tuberculosis serum IGRA test (QuantiFERON® TB Test or T SPOT.TB®) performed within 3 months prior to screening can be accepted.
o. X-rays of the lumbar and cervical spine, lateral views; to be performed at screening only if there is no image performed within 24 months prior to screening; at visit "Week 108/ET" – to be performed only at or after week 84 in case of early termination. All images of the spine to be transferred to the study coordinating centre at the Charité for central reading.p. For centres and patients participating in the MRI sub-study only. At visit "Week 108/ET" only to be performed at or after week 84 in case of early termination.

Proft F, *et al. Ann Rheum Dis* 2024; 83:599–607. doi: 10.1136/ard-2023-224699

4 INTRODUCTION

4.1 Prevalence and Clinical Manifestations of Ankylosing Spondylitis

Ankylosing spondylitis (AS) is a chronic inflammatory disease of unknown aetiology with primary involvement of the axial skeleton (sacroiliac joint [SIJ] and spine), starting in most of the cases in subjects under 45 years of age and with a strong association with the MHC class I antigen HLA-B27, which is positive in 90-95% of the patients. AS patients can develop peripheral joint and entheses manifestations, as well as extra-articular manifestations such as anterior uveitis, psoriasis and inflammatory bowel disease. The prevalence of AS is estimated to be between 0.1 and 1.4%[3]. The disease is characterized by the presence of active inflammation in the SIJ and the spine, which manifests as pain and stiffness, and by excessive new bone formation (leading to the development of syndesmophytes and ankylosis in the same areas). This will produce in up to 40% of the patients a significant functional impairment[4] and may occur with a close relationship between the grade of impairment and the duration of the disease[5]. At the same time, the disease has a relevant socio-economic impact due to disability and chronic therapies including biological drugs, generating high costs[6 7]. Reduction of clinical burden and prevention of disability can probably be best achieved by early and adequate treatment targeting for both inflammation and new bone formation.

4.2 Current Treatment of Ankylosing Spondylitis

According to the ASAS/EULAR recommendations, the first-line therapy for patients with AS are NSAIDs, including selective COX-2 antagonists, along with education and continuous exercise/physiotherapy[8]. Therapy with conventional DMARDs such as methotrexate, leflunomide, and sulfasalazine may have some beneficial effect in patients with peripheral joint involvement, but in general are not effective for the treatment of axial involvement[9-11]. For those patients who had a poor response to NSAIDs, contraindications or intolerance for NSAIDs, the only effective treatment currently available is the anti-TNF therapy[8 12]; very recently secukinumab, a monoclonal antibody against interleukin(IL)-17, has been approved in the EU for the treatment of active AS based on the positive results of two phase III studies[13].

Proft F, *et al. Ann Rheum Dis* 2024; 83:599–607. doi: 10.1136/ard-2023-224699

4.3 Evidence for disease modification in Ankylosing Spondylitis

4.3.1 NSAIDs

There is some evidence that NSAIDs, in particular Celecoxib, might possess not only a symptomatic efficacy but also disease modifying properties in AS, retarding the progression of structural damage (syndesmophytes and ankylosis) in the spine if taken continuously[14]. That might be explained by a direct inhibitory effect on osteoblast genesis and activity[15]. This effect was especially evident in AS patients with elevated CRP[16], which is also considered a risk factor for radiographic spinal progression in AS[17]. The data from the GESPIC study (a non-interventional observational cohort) showed a similar protective effect against radiographic spinal progression in those patients who had high NSAIDs intake (defined as >50% of the maximum recommended dose) and who were at high risk for radiographic spinal progression (presence of syndesmophytes and/or elevated CRP) at baseline[18]. For diclofenac, however, such effect was not proven in the recently published ENRADAS trial (another study supported by BMBF[19]); this might be explained by the lack of COX-2-selectivity of the drug.

4.3.2 TNF blocker

TNF-blocker agents are used as a second line therapy in patients with AS, if NSAIDs demonstrate no or insufficient clinical effect and have until now the best effect on inflammation and signs and symptoms[12]. Despite good clinical efficacy on inflammatory aspects, TNF-blockers are not able to retard radiographic spinal progression in AS over a period of two[20-22] or four[23] years.

4.3.3 Combination of NSAIDs and TNF blocker

Many of the patients with AS treated with a TNF-blocker discontinue their NSAIDs due to good symptom control with the anti-TNF agent (including the 5-year GO-RAISE pivotal study on the efficacy of Golimumab in AS). Therefore it has not been possible until now to answer the question of the impact of a combined therapy (TNF blocker and NSAID) on radiographic spinal progression.

It is crucial to clarify whether adding an NSAID (especially a COX-2 selective one) to TNF-blocker treatment is able to stop or reduce radiographic progression, especially in patients at high risk (i.e. with elevated CRP and/or with already present syndesmophytes). The current trial is aimed to determine the effect of the NSAID Celecoxib on radiographic spinal progression in patients treated with TNF-blockers.

Proft F, et al. Ann Rheum Dis 2024; 83:599–607. doi: 10.1136/ard-2023-224699

If such an effect exists, a benefit of retardation of radiographic spinal progression should be weighed against possible risks related to continuous NSAID therapy. Retardation of radiographic spinal progression shall have a beneficial effect on the long-term functional outcome and spinal mobility in AS patients. In case of a positive result of the trial, an optimized treatment strategy might help to avoid disability related to functional limitations caused by spinal structural damage in AS.

Proft F, *et al. Ann Rheum Dis* 2024; 83:599–607. doi: 10.1136/ard-2023-224699

5 STUDY OBJECTIVE

The objective of this study is to evaluate the impact of treatment with NSAIDs when added to anti-TNF therapy vs. anti-TNF therapy alone on progression of structural damage in the spine over two years in patients with AS.

5.1 Primary Endpoint

- Radiographic spinal progression measured by the change of the modified Stoke Ankylosing Spondylitis Spine Score (mSASSS) over two years of therapy.

5.2 Secondary Endpoints

Efficacy

- New syndesmophyte formation or progression of existing syndesmophytes after 2 years of treatment.
- Improvement of disease activity, function, axial mobility and quality of life measures at Week 12 and Week 108 in comparison to baseline according to:
  - BASDAI
  - ASDAS
  - CRP and erythrocyte sedimentation rate (ESR)
  - Bath AS Functional Index (BASFI)
  - Bath AS Metrology Index (BASMI) and chest expansion
  - Global assessment (patient/physician), general pain and nocturnal pain on the numeric rating scale (NRS)
  - ASAS Health Index
  - Physician Acceptable Symptom State (PhASS)
  - Patient Acceptable Symptom State (PASS)
  - Percentage of subjects who achieve an ASAS20, ASAS40, ASAS partial remission, BASDAI50 and ASDAS in comparison to baseline;
- Change of soluble and cellular biomarkers of inflammation and bone / cartilage turnover at week 108 in comparison to baseline and their relevance for the prediction of radiographic progression.
- Change of the enteric microbiome profile at week 108 in comparison to baseline.
- Change of osteitis score and scores for the chronic post-inflammatory changes in the spine and sacroiliac joints (SIJ) on magnetic resonance imaging (MRI) by Berlin MRI

scoring method at week 108 in comparison to baseline – for the MRI sub-study patients only.

Safety

- Number of AE and percentage of patients experienced AE, serious AE and events of interest: infections, malignancies, gastrointestinal events (ulceration, bleeding, perforation, gastric outlet obstruction), thrombotic cardiovascular events (myocardial infarction, stroke, pulmonary artery embolism, peripheral arterial of venous thrombosis), renal impairment (creatinine > 1.8 mg/dl or increase > 0.5 mg/dl vs. Baseline) and hepatic deterioration (AST, ALT, AP or GGT > 5-fold ULN). A safety follow-up visit will be performed 3 months after the discontinuation of the study treatment (either at Week 108 or last study visit in case of early discontinuation) in order to address possible long-term safety issues with special regard to continuous use of NSAIDs.

Proft F, *et al. Ann Rheum Dis* 2024; 83:599–607. doi: 10.1136/ard-2023-224699

6 INVESTIGATIONAL PLAN

6.1 Study Design

The study duration includes two phases (Figure 1):

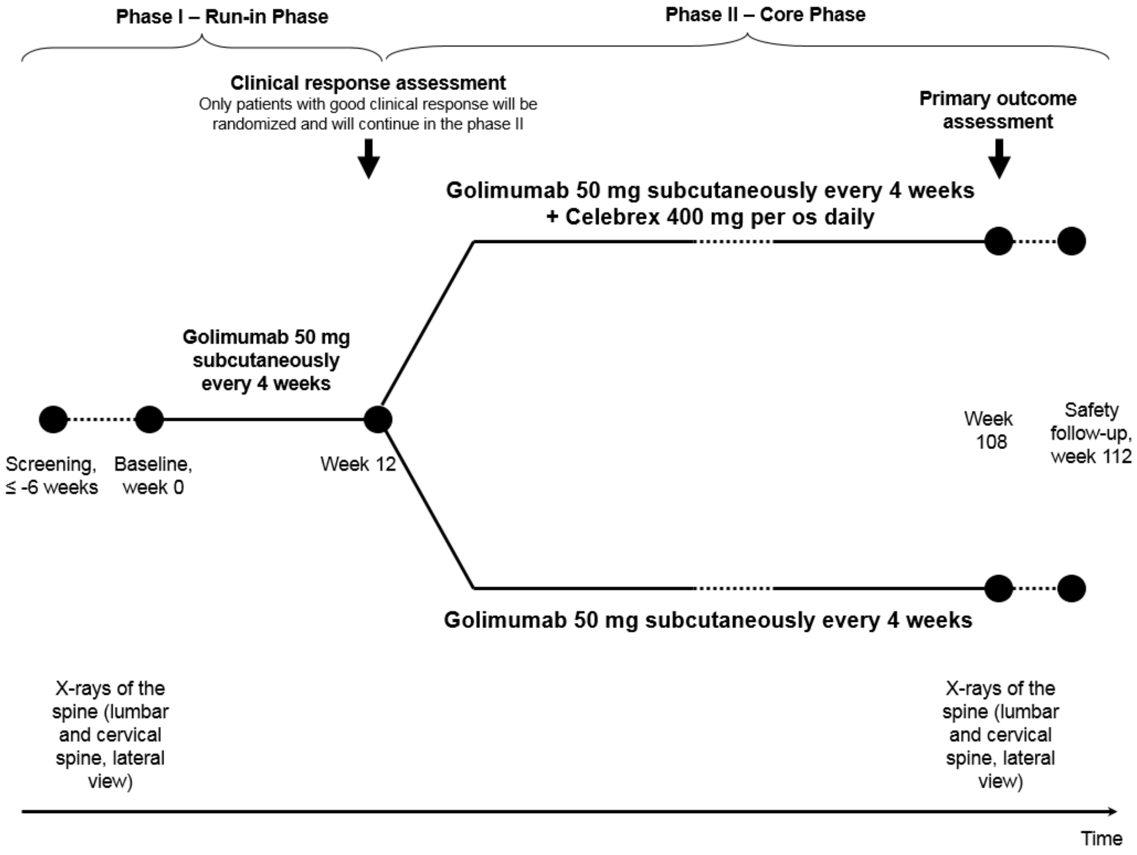

- **<u>Phase I (Run-in Phase):</u>** It includes a screening period of ≤6 weeks followed by a 12-week period where all patients receive treatment with Golimumab 50 mg SC every 4 weeks.

  <u>Screening:</u> Patients will be assessed for study eligibility and will undergo procedures outlined in the visit schedule. During this period a standard chemoprophylaxis of tuberculosis might be initiated if appropriate prior to the first injection of Golimumab.

  Subjects, who were initially regarded as screen-failures for the study, may be permitted to rescreen once following signing re-consent and require a repeat of the screening procedures.

Proft F, *et al. Ann Rheum Dis* 2024; 83:599–607. doi: 10.1136/ard-2023-224699

Baseline: eligibility assessment and start of Golimumab 50 mg SC treatment.

Visits Week 2, 4 and 8: safety assessment according to the recommendations for anti-TNF treatment.

- **Phase II (Core Phase):** it is composed by a 96-week controlled treatment period and a safety follow-up visit at week 112.

  Visit at Week 12: assessment of clinical response (achievement of at least 2 absolute points on a scale from 0-10) after 12 weeks of TNF-Blocker treatment in the phase I. Only patients who achieve a good clinical response will be included and randomized 1:1 to receive Golimumab 50mg SC every 4 weeks alone or in combination with Celecoxib 400mg per os daily.

  Visits Week 24, 36, 48, 60, 72, 84, and 96: safety assessment according to the current recommendations for anti-TNF therapy (every 3 months); clinical disease activity assessment.

  Visit at Week 108 or Early Termination: After the last Golimumab injection at Week 104 or in case of early termination (ET, only in patients who were randomised for further therapy at week 12 of the study).assessment of the primary (radiographic progression) and secondary end-points including safety assessment. A second set of X-rays of the spine and a second MRI of spine and sacroiliac joints (for patients participating in the MRI sub-study) are only to be taken, if the patient reached at least the Week 84 time point.There will be a follow-up period of 4 weeks with a safety follow-up visit (phone call) for the collection of additional safety information.

  Visit at Week 112 / Follow-up (FU):

- Visit time windows: Study visits from 3 to 6 will have a time window of ±7 days, and the study visits 7 to 15 – a time window of ±14 days. If a subject has an out of window visit the next visit should occur according to the original schedule based on the date of the baseline visit (Visit 2 / Week 0).

6.2 Justification of Design aspects

6.2.1 Control(s) / Comparator(s)

The core phase of the study compares the effect of TNF-blocker alone vs. adding an NSAID to the anti-TNF therapy on the radiographic progression in patients with active AS. For NSAIDs, Celecoxib was chosen as preferred NSAID among the others because up to date

Celecoxib is the only NSAID that has shown an inhibitory effect on radiographic spinal progression in AS[14 16]. Furthermore, there has been some evidence that Celecoxib might have a specific effect on osteoblast metabolism, not shared by the other NSAIDs[24 25]. These data justified, therefore, the choice of Celecoxib as a study drug.

For the TNF-blockers, we do not expect any differences regarding their inhibitory effect on radiographic spinal progression. Golimumab was selected based on the availability; in general, a similar efficacy of all anti-TNF-blockers can be assumed.

In the Golimumab-combined-Group, patients will be treated with a full therapeutic dose (400 mg/d)[26] of Celecoxib and Golimumab 50 mg every 4 weeks. For the control group – Golimumab-Monotherapy-Group (they will receive Golimumab 50 mg every four week alone), patients and physicians will be encouraged to discontinue NSAIDs at week 12 and treat patients with analgesics (not NSAIDs) if necessary. However, in order to increase acceptance of this study, adding NSAIDs intake on demand as a rescue medication will be allowed in the control group if demanded by patient or physician (as it was performed on the control group of the study cited above[14]). In the case of rescue therapy with an NSAID in the monotherapy group not more than 50% of the maximal recommended dose[26] will be permitted, but under no circumstance Celecoxib should be taken in the control group.

6.2.2 Dose, Mode, Duration and Scheme of Intervention

In the phase I of the study, patients with clinical indications for anti-TNF therapy (high disease activity despite treatment with NSAIDs – first line therapy) will be treated for 12 weeks with an approved dose of the TNF-blocker Golimumab 50mg SC every 4 weeks. During this period, patients shall take their NSAID in a stable dose during the first 12 weeks of the study. Any change in the dose or of the intake regimen is allowed only for a safety reason as judged by the investigator. After 12 weeks of treatment, good clinical responders will be randomized to receive treatment with Golimumab (50 mg SC every 4 weeks) and Celecoxib in full therapeutic dose (400 mg per os daily) ***or*** Golimumab (50 mg SC every 4 weeks) alone for another 96 weeks in order to assess the course of radiographic progression of the spine.

Currently, 2 years is the standard minimal duration of the study aimed at the assessment of radiographic spinal progression in ankylosing spondylitis[8].

In the combined treatment group, a Celecoxib interruption of max. 4 weeks duration will be allowed if medically justified (i.e., intolerability / side effects associated with the full NSAID

dose); a dose reduction to 200 mg/d is possible for better acceptance or safety issues (due to the discretion of the investigator), the reason for dose reduction has to be documented.
In the Golimumab-monotherapy arm, rescue medication with simple analgesics or opioids for pain and stiffness control should be preferred, in case of insufficient efficacy NSAIDs in a dose of ≤50% of the maximal recommended dose could be used for a short-time period, but under no circumstance Celecoxib should be taken in the control group.

6.3 Study Population

A total of 170 patients with active AS fulfilling the modified New York criteria who have had an inadequate response to ≥2 NSAIDs will be enrolled in the study. It is expected that 100 patients will achieve a good clinical response to Golimumab at Week 12 and will be randomized to the core study phase.

6.3.1 Inclusion Criteria

Phase I of the study:

1. Age ≥18 years.
2. Definite diagnosis of AS according to the modified New York criteria1.
3. History of an inadequate response to adequate therapeutic trials with NSAID´s,defined as at least two NSAID´s over a 4-week period in total at maximum recommended or tolerated anti-inflammatory dose
4. Active disease as defined by a BASDAI value of ≥4.0 at screening and baseline.
5. Presence of at least one of the following risk factors for radiographic spinal progression:
   - Elevated CRP (>5mg/l) at screening and the absence of reasons for elevated CRP other than AS;
   - Presence of ≥ 1 syndesmophyte on prior X-rays of the spine.
6. Subject is a candidate for anti-TNF therapy based on the investigator’s opinion.
7. Subject is able and willing to give a written informed consent and comply with the requirements of the study protocol. Only patients who give written informed consent will be included in the trial.
8. If female: either unable to bear children (postmenopausal for at least 1 year or surgically sterile) or is willing and able to practice a reliable method of contraception throughout the study and 6 months after the last dose of study drug. A highly effective method of birth control is defined as those which result in a low failure rate (i.e. less than 1% per year) when used consistently and correctly such as implants, injectables, combined oral contraceptives or a vasectomised partner (at least 1 year prior to enrolment). In questionable cases of menopausal status a blood sample with simultaneous levels of

follicle stimulating hormone (FSH) above 40 U/l and estradiol below 30 pg/ml is confirmatory. The results of the pregnancy test performed at Screening must be negative.

9. If on NSAIDs: the dose must be stable for at least 2 weeks prior to baseline.
10. If on analgesics: the dose must be stable for at least 2 weeks prior to baseline.

Phase II of the study:

Achievement of an improvement of ≥2 absolute points (on a 0-10 scale) of the BASDAI after 12 weeks of Golimumab treatment in Phase I

6.3.2 Exclusion Criteria

1. For female subjects: pregnancy or lactating.
2. Subjects with chronic inflammatory articular disease other than SpA or systemic autoimmune diseases, e.g. systemic lupus erythematosus, Sjögren´s syndrome, rheumatoid arthritis. *Other manifestations of SpA such as psoriasis, uveitis, peripheral arthritis are not regarded as exclusion criteria*.
3. History of inadequate response (primary non-response) to previous anti-TNF therapy. Patients who discontinued previous anti-TNF therapy or other biologics for reasons other than primary non-response may participate in the study if anti-TNF therapy is considered to be suitable in the opinion of investigator. This prior therapy must have been terminated at least 3 months (in case of TNF blockers or secukinumab) or 5 half-lives in the case of other biologics prior to Baseline visit.
4. Treatment with any other investigational drug within 3 months or 5 half-lives of the drug (whichever is longer) prior to Baseline visit.
5. History of intolerability or hypersensitivity reaction to Golimumab.
6. History of intolerability or hypersensitivity reaction to Celecoxib or Sulphonamides.
7. Previous reactions of asthma, urticaria or other severe hypersensitivity/reactions after intake of acetylsalicylic acid or other NSAIDs.
8. Known hypersensitivity to any component of the study medications.
9. Treatments with DMARDs other than methotrexate or sulfasalazine within 4 weeks prior to baseline (in case of leflunomide either 8 weeks or 4 weeks with a standard cholestyramine wash-out).
10. The presence of total spinal ankylosis.
11. Treatment with intravenous, intramuscular or intraarticular/periarticular steroids within 4 weeks prior to Baseline Visit, treatment with oral steroids in a dose of >10 mg

Proft F, *et al. Ann Rheum Dis* 2024; 83:599–607. doi: 10.1136/ard-2023-224699

(prednisolone equivalent) per day.

12. Any active current infection. History of recurrent clinically significant infection (suggestive for primary or secondary immunodeficiency). Infections requiring treatment with antibiotics within 4 weeks prior to baseline.
13. Current clinical signs and symptoms suggestive for tuberculosis.
14. Positive tuberculosis IGRA serum test and abnormal chest x-ray (both at screening, maybe accepted if performed within 3 months prior to screening) suggestive for past or present tuberculosis (positive x-ray). *Patients with a positive Tuberculosis IGRA serum test but negative chest x-ray and without clinical symptoms suggestive for tuberculosis may participate in the study after initiation of standard prophylactic anti-tuberculous treatment, which has to be started 4 weeks before initiation of study medication and has to be taken for a total of 9 months.*
15. Chronic infection with hepatitis B virus (*in case of HBsAg negativity, but anti-HBc positivity, participation in the study possible after hepatologists consultation*):. (see also table in section 6.7).
16. Actual malignancies or history of malignancies with curative treatment within 5 years prior to screening, except successfully treated non-metastatic squamous-cell or basal-cell carcinoma of the cutis or carcinoma in situ of the cervix.
17. History of demyelinating disease (like multiple sclerosis; including myelitis) or neurologic symptoms suggestive of demyelinating disease.
18. History of cardiovascular events: myocardial infarction, stroke or transient ischemic attack, pulmonary embolism.
19. Diagnosis of the coronary heart disease or peripheral arterial occlusive disease; uncontrolled arterial hypertension.
20. High cardiovascular risk as assessed by the presence of >2 cardiovascular risk factors (e.g., uncontrolled hypercholesterolemia, diabetes mellitus, smoking, uncontrolled arterial hypertension).
21. History of oesophageal, gastric, pyloric channel or duodenal ulceration documented by endoscopy/radiographic examination at any time before the screening visit, or any clinically relevant gastrointestinal bleeding, perforation, or gastric outlet obstruction.
22. History of a chronic inflammatory bowel disease (Crohn’s disease or ulcerative colitis).
23. Evidence of other severe uncontrolled gastrointestinal, hepatic (serum albumin <25 mg/l or Child-Pugh-Score >10), renal, pulmonary, cardiovascular, nervous or endocrine disorders.

Proft F, *et al. Ann Rheum Dis* 2024; 83:599–607. doi: 10.1136/ard-2023-224699

24. Increased bleeding risk including therapy with anticoagulants and thrombocyte aggregation inhibitors (e.g. low-dose aspirin).
25. Chronic or acute congestive heart failure (NYHA II to IV).
26. Patients with a history of a severe psychiatric illness, which might interfere with the patient's ability to understand the requirements of the study and assessment.
27. Diagnosis of fibromyalgia.
28. Alcohol abuse or illegal drug consumption in the last 12 months.
29. Vaccination with a live vaccine within 12 weeks prior to baseline.
30. Any of the following laboratory abnormalities as detected at screening:
    - Haemoglobin < 8.5 g/dl
    - Neutrophil counts < 2.000 / µl
    - Platelet count < 125.000 / µl
    - Aspartate aminotransferase (AST) or alanine aminotransferase (ALT) or alkaline phosphatase (AP) or gamma-glutamyl-transpeptidase (GGT) >3 times upper limit of normal
    - Creatinine > 1.4 mg / dl (= 120 µmol / l).
31. Patients who are defined as “vulnerable patients”: patients who are institutionalised due to regulatory or juridical order; patients who are an employee of the investigator or study site, with direct involvement in the proposed study or other studies under the direction of that investigator or study site; family members of the employees or the investigator.
32. Any other conditions making the patient unsuitable in the opinion of the investigator for the participation in the current study.
33. <u>For patients participating in the MRI sub-study only</u>: patients with contraindications for the magnetic resonance imaging (MRI) including but not limited to: claustrophobia, seizure disorders, presence of a implanted electronic devices (e.g., heart pacemaker, insulin pump, etc.), metal implants and metal foreign bodies in the patient’s body suspected to be ferromagnetic, tattoos performed with metal-containing paints or tattoos of the large skin areas.

6.4 Prior and Concomitant Therapy; Prohibited Medications

All medications during the entire study period should be recorded. All drug therapies for AS should be stable prior to the first Golimumab administration (as stated in the inclusion/exclusion criteria). Changes in concomitant therapy required for the treatment of

Proft F, et al. Ann Rheum Dis 2024; 83:599–607. doi: 10.1136/ard-2023-224699

adverse events are allowed. All changes in the concomitant therapy must be recorded. Allowed changes in the AS therapy are described in the section 6.5.2 "Rescue Treatments".

The following medications are prohibited during the study:

- Systemic steroids in a dose >10 mg (prednisolone equivalent) per day,
- DMARDs other than methotrexate or sulfasalazine,
- Biologics other than the study drug Golimumab,
- Any investigational drugs of chemical or biological nature,
- Live vaccines (during the study and 60 days after the last drug administration).

6.5 Study Treatment

6.5.1 Treatments Administered

Included patients will receive Golimumab 50 mg administered subcutaneously by the study personnel or self-administered during the study visit at baseline (week 0), Week 4, 8 and 12. The following dosages will be self-administered by the patient as SC injections every 4 weeks. In the Phase II, subjects included in the Golimumab-Combined-Arm will receive in addition Celecoxib 400mg per os (2 capsules containing 200 mg each) daily.

If the administration of the study drug Golimumab should not be performed in the opinion of the investigator at the planned date due to AE (e.g. infections or clinically significant laboratory abnormalities), the administration should be possible after resolution of the AE, but not later than 14 days after the scheduled injection date. If Golimumab administration is not possible within this time window, this injection should be skipped and next injection has to be done according to the original schedule. Treatment with Celecoxib could be interrupted if necessary for up to 4 weeks.

6.5.2 Rescue Treatments

Only Golimumab responders will be included in the phase II of the trial, therefore substantial problems with insufficient efficacy of treatment or non-compliance during this phase are not expected. Nonetheless, there will be the possibility of rescue treatment for all patients experiencing pain and stiffness:

1) Analgesics without anti-inflammatory capacity (e.g. paracetamol, metamizole or tramadol) in usual therapeutic doses, which will be prescribed by the rheumatologist.

Proft F, et al. Ann Rheum Dis 2024; 83:599–607. doi: 10.1136/ard-2023-224699

2) For patients in the Golimumab-monotherapy-arm and insufficient response to analgesics, on-demand use of NSAIDs up to 50% of the maximum therapeutic dose (i.e. Ibuprofen up to 1200 mg/d; see table below) will be allowed; but, underno circumstance Celecoxib should be taken in the control group.

In both arms of the phase II a proton pump inhibitor (PPI) in standard dose could be prescribed by the rheumatologist as needed, reflecting standard of care for treatment or prevention of gastric side effects of NSAID.

Table. Approved maximal daily doses of the most common NSAIDs in Germany.

NSAID	Approved maximal daily dosage in Europe (Germany), normally for arthritis	Label for AS in Europe (Germany)
Ibuprofen	2400 mg (1-3 divided doses)	Yes
Indomethacin	150 mg (1-3 divided doses)	Yes
Ketoprofen	200 mg (1-2 divided doses)	Yes
Naproxen	1000 mg (1-2 divided doses)	Yes
Piroxicam	20 mg (1-2 divided doses)	Yes
Meloxicam	15 mg (once daily)	Yes
Diclofenac	150 mg (1 single or 2-3 divided doses)	Yes
Aceclofenac	200 mg (1-2 divided doses)	Yes
Etoricoxib	90 mg (1 single dose)	Yes

6.5.3 Drug supplies, Packaging and Labelling

MSD Sharp & Dohme GmbH (Haar/Germany) will provide the TNF-blocker (Golimumab) as an open-label supply in carton boxes (Simponi®). Each carton box will contain one pre-filled syringe with Golimumab 50mg / 0.5 ml. On the product label, the study identifier, the product identity and strength, batch number, directions for use, list of excipients, storage conditions and expiration date will be indicated.

Proft F, *et al. Ann Rheum Dis* 2024; 83:599–607. doi: 10.1136/ard-2023-224699

Celecoxib (Celebrex® manufactured by Pfizer Deutschland GmbH) will be provided as an open-label supply in carton boxes containing 100 capsules á 200mg. On the product label, all details will be indicated too.

6.5.4 Storage, Disposition and Dug Accountability

Golimumab pre-filled syringes must be stored locked in a secure place, protected from light at 2° to 8°C. Study medication must not be frozen at any time. A storage temperature log is to be maintained on a daily basis. In case of temperature regiment deviations the drug should be quarantined and not dispended until Sponsor deems the medication as acceptable.

Celecoxib capsules are to be stored at room temperature, no temperature log is needed.

The investigator or designee should verify that study drug supply is received intact at the appropriate temperature and in the correct amount. For regulatory requirements (GCP), the site has to maintain 1.) a “Site Drug Accountability Log”, which includes information on the date of receipt, box count, the batch number, date dispensed, subject number and identification of the person who dispensed the drug and 2.) an individual “Patient Drug Accountability Log” including information on date and box count of dispensed and count of received (empty/used) syringes or pill blisters. The investigator is responsible for accounting of the used and unused study drug. Expired study drug must not be administered.

All returned study medication (e.g. empty syringes, broken pill containers/blisters) will be collected, documented and disposed by the study site. At every study visit complete new boxes / containers of study medication will be handed to the patient.

The study drug must not be used for other reasons than the current study and only in patients who were enrolled in the study and gave a written informed consent. Celecoxib from this study is only to be handed to patients randomized into the combination arm of the Phase II, but not as rescue medication for patients in the Golimumab only arm.

6.5.5 Treatment Compliance

The subject him/herself or the qualified study staff will administer all injections of study drug. In order to document compliance with the treatment, administration (dates and times) of the study drug will be recorded in the patient’s chart and in the Drug Accountability log. Subjects will be instructed to return all drug containers / syringes from Golimumab and blisters from Celecoxib (even empty) to the study site.

6.6 Study Discontinuation

6.6.1 Discontinuation of Individual Subjects

A subject may withdraw from the study at any time. The investigator may discontinue the subject participation due to adverse events, safety concerns or subject's failure to comply with the protocol.

Subjects will be withdrawn from the study if one of the following occurs:

- The subject requests withdrawal from the study.
- The subject does not achieve a good clinical response (improvement of at least ≥2 absolute points on a 0-10 scale of BASDAI after 12 weeks of treatment with Golimumab in the phase I.
- The female subject of child-bearing potential does not perform effective contraceptive methods or becomes pregnant during the study.
- The subject is diagnosed with a malignancy or lymphoproliferative disorder.
- The subject develops allergic reaction to / intolerance of the study drug.
- The subject is diagnosed with multiple sclerosis or demyelinating disease.
- The subject develops a lupus like syndrome.
- Two consecutive Golimumab injections were not performed.
- For patients in the combination arm: Study treatment with Celecoxib was interrupted for more than 4 weeks.
- Introduction of a prohibited medication after the subject started the study drug, when continuation of the study therapy would place the subject at risk as defined by the investigator.
- The subject receives a live vaccine during the study period.
- Major surgical intervention during the study period.
- The subject is diagnosed with active tuberculosis, opportunistic infection or any other severe infection including acute Hepatitis B or HIV.
- Subject is non-compliant with anti-tuberculous treatment (if this medication is required).

Proft F, et al. Ann Rheum Dis 2024; 83:599–607. doi: 10.1136/ard-2023-224699

- Any AEs, including clinically significant laboratory abnormalities, when continuation of the study therapy would place the subject at risk as defined by the investigator.
- Any other reason at the investigators discretion to ensure the well-being of the subject.

In case of premature discontinuation of the study procedures outlined in the assessment schedule for the “Early Termination Visit” should be performed within 4 weeks after last dose of study drug. The reason for the discontinuation must be documented. After study discontinuation the patients will be treated according to available standards of care.

6.6.2 Discontinuation of the Entire Study

Both the sponsor and the investigator reserve the right to terminate the study at any time. In this case, both parties will arrange the procedures on an individual study basis after review and consultation. Advance notice is not required by either party if the study is stopped due to safety reasons.

Criteria for discontinuation of the entire study:

- Negative benefit/risk assessment due to new information
- Decision of the ethical committee or of the federal authority
- Certain circumstances, such as lack of drug supply from the third parties, making continuation of the study impossible.

When terminating the study, the investigator will assure that adequate consideration is given to the protection of the patient’s interests. In case of premature termination of the study all collected data has to be analysed and a report has to be written. The sponsor (Charité) has the responsibility by law (AMG / GCP) to inform the federal regulatory authority, the ethics committees and other possibly related authorities within 15 days, giving detailed reason for the premature termination.

6.7 Study Procedures

The study procedures are outlined in the assessment schedule. All procedures will be documented in the patient’s charts and in the patient’s case report form (CRF).

Proft F, *et al. Ann Rheum Dis* 2024; 83:599–607. doi: 10.1136/ard-2023-224699

Informed Consent

An Independent Ethics Committee (IEC) / Institutional Review Board (IRB) approved informed consent will be reviewed, signed and dated by the subject, before any study-related procedures are undertaken.

Inclusion/Exclusion criteria

Subjects should meet all inclusion and none of the exclusion criteria at both screening and Baseline Visits in order to be eligible for the study.

Demographics

Patient's age and gender will be collected at the Screening Visit.

Medical/Surgical history

A complete medical and surgical history including history of articular (inflammatory back pain, peripheral arthritis, enthesitis, dactylitis) and extraarticular manifestations (uveitis, psoriasis, inflammatory bowel disease), and spondyloarthritis (SpA) family history will be collected during the Screening Visit and updated at Baseline Visit and throughout the study as applicable. Smoking status will be collected at different time points. The certificate of vaccination ("Impfausweis") of the patient shall be checked at screening visit, recommended vaccinations according to local guidelines should be performed prior to Baseline; in case of live vaccines (e.g. measles, rubella, varicella) start of golimumab shall not be prior to 12 weeks after vaccination (see Exclusion Criterion 29).

Prior medication for SpA

As part of in-/exclusion criteria.

Concomitant medication

All concomitant medications will be collected at screening and updated during following visits.

Vital signs, weight, height

Vital signs including heart rate, blood pressure (both measured in sitting position after resting for at least 5 minutes), weight and height will be obtained as outlined in the assessment schedule.

Physical examination

A routine physical examination will be performed as outlined in the assessment schedule.

Proft F, *et al. Ann Rheum Dis* 2024; 83:599–607. doi: 10.1136/ard-2023-224699

Axial mobility measurement

Axial mobility measurements will include BASMI [27] and chest expansion measurement [28] and will be performed as outlined in the assessment schedule.

Laboratory analyses

Blood chemistry, haematology, C-reactive protein, erythrocyte sedimentation rate, serum pregnancy test (female subjects), hepatitis B (HBs-Ag and anti-HBc) serology, and urinalysis will be performed as outlined in the assessment at the respective local laboratory of the study site as a part of clinical routine in patients treated with TNF blockers. At screening, blood analysis includes also Quick-Test, International Normalized Ratio (INR) and activated Partial Thromboplastin Time (aPTT) to recognize patients with high bleeding risk.

In patients with positivity in the above mentioned tests for hepatitis B, further recommendations exist: due to small risk (1.7-5%) for HBV reactivation in “HBs Ag negative & anti HBc positive” patients, a participation of the patient in the CONSUL study is possible only after hepatologist assessment (see Table 1).

Table 1. How to proceed with positive Hepatitis B virus tests

HBs Antigene	Anti HBc Antibodies	Recommendation
negative	Negative	Study participation possible
Positive	pos./neg.	Exclusion criterion in CONSUL, general recommendation for referral to a hepatologist
Negative	Positive	Referral to hepatologist:<br>Testing for HB virus load (PCR):<br>-negative – study participation possible, “watchful waiting”<br>-positive – anti TNF therapy might be discussed after initiation of pre-emptive HBV therapy

Analyses for research purposes (blood tests for biomarkers of inflammation, bone / cartilage turnover, stool microbiome) will be performed centrally at the Charité Berlin and corresponding laboratories.

Tuberculosis screening

All patients will undergo an IGRA tuberculosis serum test (QuantiFERON®-TB Gold In-Tube test or T.SPOT TB®) and a chest x-ray (tests performed within three months prior to screening can be accepted) at Screening Visit. The history of active or latent tuberculosis infection as well as physical examination will be collected in order to reveal signs and

Proft F, *et al. Ann Rheum Dis* 2024; 83:599–607. doi: 10.1136/ard-2023-224699

symptoms suggestive for tuberculosis. Patients with clinical or radiological signs suggestive for tuberculosis will be excluded from the study participation. Subjects with a negative tuberculosis serum test are eligible to continue with the screening procedures.

In case of a positive tuberculosis serum test but no radiological or clinical signs suggestive for tuberculosis the subject might participate in the study upon initiation of the prophylactic anti-tuberculous treatment (e.g., isoniazid 300 mg daily initiated 4 weeks prior to the first administration of Golimumab and continued for a total of 9 months in combination with pyridoxine).

Electrocardiography (ECG)

A 12-lead ECG should be performed in all patients, who are considered by the investigator to be at risk of having unrecognized cardiovascular pathology (coronary heart disease, rhythm and conduction disturbances).

Patient questionnaires

Patients questionnaires (BASDAI [29], BASFI [30], Patient's Global, overall pain, nocturnal pain, ASAS Health Index[31], Patient Acceptable Symptom State PASS[33]) represent validated tools for the assessment of disease activity, functional status, quality of life and overall well-being in AS; they will be completed according to the assessment schedule.

Physician's Global and Physician Acceptable Symptom State

Represent global assessment of disease activity and patient's symptom state by the investigator will be performed according to the assessment schedule.

X-rays of the spine

X-rays of the lumbar and cervical spine (lateral views) will be performed during screening only if there were no image performed within the previous 24 months. Those images are central for the conduct of the study (primary outcome; inclusion criterion #5b and exclusion criterion #10). Quality of the x-rays should allow this assessment, i.e., images should be available in electronic form or as x-ray film (no paper printouts). If no images fulfilling these criteria are available, X-rays of the cervical and lumbar spine should be performed during the screening period.A second set of X-rays of lateral cervical and lumbar spine must be performed at Week 108, or in case of an early termination (ET) if the patient reached at least Week 84..

Magnetic resonance imaging (MRI) of spine and sacroiliac joints

Proft F, *et al. Ann Rheum Dis* 2024; 83:599–607. doi: 10.1136/ard-2023-224699

MRI of the spine and sacroiliac joints will be performed during screening (before first injection of Golimumab), and at Week 108 (or in case of an early termination (ET) if the patient reached at least Week 84)) in a sub-study with patients of study sites in Berlin & Brandenburg having no contraindication for this investigation. No contrast agents will be applied.

Golimumab administration

Golimumab 50 mg will be administered subcutaneously by the qualified study personnel or self-administered by the subject during the study visits at baseline and then every 4 weeks until Week 108 – last injection will be at week 104 (for non-responders dropping out of study at week 12 – last injection at week 8).

Proft F, *et al. Ann Rheum Dis* 2024; 83:599–607. doi: 10.1136/ard-2023-224699

7 SAFETY CONSIDERATIONS

7.1 Definition and Reporting of Adverse Events

An adverse event (AE) is defined as any new undesirable medical occurrence in a clinical investigation subject / patient having received at least one dose of a study drug; this does not necessarily have to be a causal relationship with the study treatment. An AE can therefore be any unfavourable and unintended sign (including an abnormal laboratory finding, for example), symptom, or disease during the time course of the study.

Such an event can result from use of the drug as stipulated in the trial or labelling, as well as from accidental or intentional overdose, drug abuse, or drug withdrawal. Any worsening of a pre-existing condition or illness is considered an AE. Worsening in severity of a reported AE should be registered as a new AE. Laboratory abnormalities and changes in vital signs are considered to be AE only if they result in discontinuation from the study, needs therapeutic medical intervention and/or if the investigator considers them to be an AE.

Following the subject's written consent to participate in the study, all serious AEs (SAE) should be collected. All other AEs should be collected throughout the treatment period from Week 0 until Week 108 and 4 weeks thereafter in the "Safety-Follow-up Visit" at Week 112.

If AEs occur, the subject should be followed until resolution or stabilization, and reported as serious AEs if the AE becomes serious. This also applies to subjects experiencing AEs that cause interruption or discontinuation of the investigational product, or those experiencing AEs that are present at the end of their participation in the study; such subjects should receive post-treatment follow-up as appropriate. If an on-going AE changes in its severity or in its perceived relationship to study drug, a new AE entry for the event should be completed.

A pre-planned elective surgery/procedure scheduled to occur during the study will not be considered an AE if the surgery/procedure is being performed for a pre-existing condition and no worsening of the pre-existing condition occurred during the study.

All identified AEs must be recorded and described on the appropriate AE section of the CRF. If known, the diagnosis of the underlying illness or disorder should be recorded, instead of the individual symptoms. The following information should be captured for all AEs: date of onset and resolution, severity of the event (see definitions), investigator's opinion of the relationship to investigational product (see definitions), treatment required for the AE, cause of the event (if known), and information regarding resolution/outcome. The following categories and definitions of severity should be used:

Proft F, et al. Ann Rheum Dis 2024; 83:599–607. doi: 10.1136/ard-2023-224699

- Mild (Grade I) - awareness of event but easily tolerated.
- Moderate (Grade II) - discomfort enough to cause some interference with usual activity.
- Severe (Grade III) - inability to carry out usual activity.
- Very severe (Grade IV) - debilitating significantly incapacitates subject despite symptomatic therapy.

The following categories and definitions of causal relationship to study drug should be used:

- No related: there is no causal relationship (in the opinion of the investigator) between the AE and use of the study drug.
- Doubtful: there are other factors such as concomitant disease, medications or the relationship in time, suggesting that a causal relationship is unlikely.
- Possible: the AE might be due to the use of the drug. Other factors such as concomitant drug or disease are inconclusive. The relationship in time is reasonable; therefor, the causal relationship cannot be excluded.
- Probable: there is a reasonable causal and temporal relationship between the study drug and the AE. Other reasons are less likely.
- Very likely: there is a reasonable causal and temporal relationship between the study drug and the AE. The AE is described as a side effect of the study drug and cannot be reasonably explained by an alternative explanation. The relationship in time is very suggestive (e.g. it is confirmed by dechallenge and rechallenge).

7.2 Definition and Reporting of a Serious Adverse Event

7.2.1 Definition of a Serious AE

A serious AE is any untoward medical occurrence that at any dose (based on ICH and EU Guidelines on Pharmacovigilance for Medicinal Products for Human Use):

- Results in death
- Is life-threatening (defined as an event in which the subject or patient was at risk of death at the time of the event; it does not refer to an event which hypothetically might have caused death if it were more severe)

Proft F, *et al. Ann Rheum Dis* 2024; 83:599–607. doi: 10.1136/ard-2023-224699

- Requires inpatient hospitalization or causes prolongation of existing hospitalization
- Results in persistent or significant disability/incapacity
- Is a congenital anomaly/birth defect

Medical and scientific judgment should be exercised in deciding whether expedited reporting is also appropriate in situations other than those listed above. For example, important medical events may not be immediately life-threatening or result in death or hospitalization but may jeopardize the subject or may require intervention to prevent one of the outcomes listed in the definition above (e.g., suspected transmission of an infectious agent by a medicinal product is considered a serious adverse event). Any adverse event is considered a serious adverse event if it is associated with clinical signs or symptoms judged by the investigator to have a significant clinical impact.

7.2.2 Reporting a Serious AE

All serious AEs (SAE) whether related or unrelated to investigational product, must be reported to the sponsor within 24 hours of being made aware of the serious AE:

Med. Klinik I, Rheumatologie
Campus Benjamin Franklin
Charité Universitätsmedizin Berlin
Hindenburgdamm 30
12203 Berlin, Germany
Tel: +49 30 8445 4414 / -2302
Fax: +49 30 8445 4149

The SAE report form should include at least the following information: study title, study centre identifier, patient number, age, sex, height and weight of the patient, date of the event onset, description of the event, concomitant diseases and medications, action taken, evaluation of the relationship with the study drug or study procedure, outcome. The first report should contain at least the following information: study title, study drug, study centre identifier, patient number, SAE description and investigator's statement on relationship to the study drug.

Collection of complete information concerning SAEs is highly important. Therefore, follow-up information which becomes available as the SAE evolves, as well as supporting documentation (e.g., hospital discharge letter, autopsy reports), should be notified to the

sponsor subsequently, if not available at the time of the initial report, and sent within 24 hours of being made aware using the same procedure as the initial SAE report.

All SAEs must be collected within the entire study period (108 weeks treatment and 4 weeks of follow-up) or at least 30 days after the last drug administration if the patient discontinues the study prematurely. In addition, any SAE occurring after this time period, which might be, in the opinion of the investigator, very likely, probably or possibly related to investigational product, will be reported.

The sponsor / principal investigator will be responsible for submitting of “Suspected Unexpected Serious Adverse Reaction (SUSAR)” reports to the Independent Ethics Committee and to regulatory authority and for writing and submitting the “Annual Safety Report” concerning safety of the included subjects.

7.3 Pregnancy

All female subjects of child-bearing age require a negative pregnancy test at trial entry. Female patients are required to use an adequate contraception method throughout the study period and at least 6 months after the last Golimumab injection (as outlined in chapter 6.3.1 “Inclusion Criteria”, paragraph 8). If any subject becomes pregnant during the study, she will be immediately withdrawn from the trial. In the case of pregnancy this must be reported on AE pages although the pregnancy per se is not considered an AE. However, the medical outcome of an elective or spontaneous abortion, stillbirth or congenital anomaly will be considered a serious AE and must be reported immediately (within 24 hours of the site becoming aware of the event). Follow-up information regarding the outcome of the pregnancy and any postnatal sequelae in the infant will be required.

7.4 Data and Safety Monitoring Committee (DSMC)

Safety data, especially SAE / SUSAR, but also cumulated AEs will be reviewed by an external Data and Safety Monitoring Committee consisting of 3 experienced rheumatologists otherwise not related with this clinical study. Meeting of the DSMC will take place at least once yearly.

8 PLANNED TREATMENT OF THE PATIENTS AFTER STUDY END

Patients will receive the standard treatment with standard care as needed throughout and after the study. After Week 108 any additional or changed treatment is left to the discretion of the investigator and the patient.

Proft F, *et al. Ann Rheum Dis* 2024; 83:599–607. doi: 10.1136/ard-2023-224699

Add-on studies or additional scientific projects linked to this study are possible. Subjects having terminated study participation or still participating may participate in these studies/projects if this is not contradictory to this protocol.

Proft F, *et al. Ann Rheum Dis* 2024; 83:599–607. doi: 10.1136/ard-2023-224699

9 OVERALL RISK/BENEFIT ASSESSMENT

9.1 Celecoxib

NSAIDs are the standard medical treatment in AS[8 34]. Given the chance that NSAIDs may inhibit structural damage[14], continuous treatment with an NSAID for a period of 2 years in addition to TNF-blocker treatment might therefore contribute to the improvement of the long-term outcome besides just symptomatic improvement, especially in subjects at risk (i.e. presence of elevated CRP and syndesmophytes). NSAIDs treatment might be particularly beneficial in patients with these characteristics as several studies indicated [16 18]. Possible risks of NSAIDs in the treatment of rheumatic diseases as AS are well described and patients with AS are at lower risk for side effects of NSAIDs including Celecoxib because of lower age and lower prevalence of comorbidities in comparison to other rheumatic conditions such as rheumatoid arthritis or osteoarthritis[35]. Generally COX-2 selective NSAIDs have a lower risk of producing severe gastrointestinal side effects than non-selective NSAIDs. Nevertheless, since the combined treatment group will be taking daily Celecoxib for 2 years, a concomitant therapy with PPI can be prescribed to reduce the risk of gastrointestinal AE (decision of the investigator).

High risk patients for experiencing cardiovascular side effects will be excluded from this study. The high risk is defined as having > 2 risk factors (e.g., uncontrolled hypercholesterolemia, diabetes mellitus, smoking, uncontrolled arterial hypertension). Medical history of myocardial infarction, recent angina pectoris, significant heart insufficiency (NYHA II or IV) as well as diagnoses of coronary heart disease and peripheral arterial occlusive disease are exclusion criteria.

Because of younger age and less comorbidities of AS patients as compared to those with rheumatoid arthritis or osteoarthritis in most of the clinical trials, we expect a low number of serious adverse cardiovascular events[19]. Two recent observational studies demonstrated that NSAIDs intake including Coxibs in AS patients is associated with no excess (or even lower) in cardiovascular risk as compared to the general population[36 37].

9.2 Golimumab

For those patients with AS and unsatisfactory response to previous NSAIDs, TNF blocking therapy (including Golimumab) is the second line therapy currently available, along with recently approved secukinumab in EU. Up to date, between 250-300.000 patients were treated with Simponi® world-wide (personal information by MSD Sharp & Dohme, spring 2016).

Proft F, et al. Ann Rheum Dis 2024; 83:599–607. doi: 10.1136/ard-2023-224699

The major potential risks of Golimumab treatment are infections, development of malignancies and hypersensitivity reactions. Recently an analysis of pooled data from the long-term extension clinical trials (up to 3 years) with patients with RA, PsA or AS receiving Golimumab has been published. Across five phase-III studies with Golimumab SC, 639 patients received placebo and 2,226 were treated with Golimumab for up to 3 years (1,249 patients were treated with Golimumab 50mg SC and 1,501 patients with Golimumab 100mg SC); a total of 1,179 subjects received Golimumab for over 156 weeks. The collected results indicate that in general Golimumab safety profile is similar to the known safety profile of other TNF α blockers.

Hypersensitivity reactions in clinical studies of Golimumab, rash and urticarial, have each been observed in < 2% of patients.

Formation of antibodies against TNF α agents has been related in different studies to a possible cause of the development of resistance or gradual drug failure and to a risk of development of hypersensitivity reaction. The risk of producing antibodies varies with each specific type of TNF-blocker, most commonly present with infliximab and adalimumab therapy[38]. Consistently in all studies performed with Golimumab, antibodies against Golimumab were detected in a small number of subjects without a clear influence on the response to therapy[39 40]; this observation suggests that the presence of anti-Golimumab antibodies does not necessarily affect the efficacy of Golimumab[41].

Based on these considerations we assume a favourable risk/benefit profile of this study. In order to minimize the overall risk to participating subjects, this protocol has inclusion and exclusion criteria appropriate to the population and proposed treatments, exclusionary screening tests, and specific follow-up safety assessments.

These criteria and assessments are also orientated to established international and national practice guidelines (EULAR[8], DGRh[42]).

Proft F, *et al. Ann Rheum Dis* 2024; 83:599–607. doi: 10.1136/ard-2023-224699

10 ETHICAL ASPECTS

10.1 Declaration of Helsinki / Good Clinical Practise (GCP)

The study will be performed according to the Declaration of Helsinki from 1996. That is the World Medical Association Recommendations guiding physicians in biomedical research involving human subjects (which was adopted by the 18th World Medical Assembly at Helsinki in 1964 (amended at subsequent meetings at Tokyo in 1975, Venice in 1983, Hongkong in 1989 and 1996 (Art. 3, Abs. 2 Guideline 2005/28/EG)).

Additionally it is the responsibility of all engaged in research on human beings to ensure that the study is performed in accordance with the international GCP standards and according to all local laws and regulations concerning clinical studies.

GCP requires that the clinical protocol, any protocol amendments, the Investigator's Brochure, the informed consent and all other forms of subject information related to the study or any other necessary documents be reviewed by and IEC/IRB. The IEC/IRB will review the ethical, scientific, and medical appropriateness of the study before it is conducted. IEC/IRB approval of the protocol, informed consent and subject information and/or advertising, as relevant, will be obtained prior to the authorization of drug shipment to a study site.

In this study no Investigator´s Brochure will be submitted, because both study drugs are approved and marketed in the indication ankylosing spondylitis in adults (according to Art. 11 REGULATION (EU) No 536/2014). Instead, the German version of the "Summary of product characteristics" ("Fachinfomation") is provided.

10.2 Patient Information and Informed Consent

The investigator will explain the nature of the study to the subject, and will answer all questions regarding this study. It is the responsibility of the investigator to obtain written informed consent from each subject participating in this study, after adequate explanation of aim, importance, anticipated benefits, and potential hazards and consequences of the study according to § 40 Abs 2 and § 40 Abs. 2a of the German drug law (AMG). Written informed consent must be obtained before any study specific procedures are performed, according to §40 Abs. 1 S. 3 Nr 3 b) and c) AMG. It must be also explained to the subject that they are completely free to refuse to enter the study or to withdraw from it at any time for any reason without incurring any penalty or withholding of treatment on the part of the investigator.

Proft F, et al. Ann Rheum Dis 2024; 83:599–607. doi: 10.1136/ard-2023-224699

By signing the consent form, the subject/patient agrees with the "unwiderrufliche datenschutzrechtliche Einwilligung" according to § 40 Abs. 2a AMG. The subject/patient also agrees to allow the monitor/auditor/health authorities to verify the collected patient data against the subject's/patient's original medical records for the purpose of source data verification.

A copy of the informed consent form will be given to the subject and the original will be placed in the subject’s medical record. An entry must also be made in the subject’s dated source documents to confirm that informed consent was obtained prior to any study-related procedures and that the subject received a signed copy.

If new safety information results in significant changes of the risk/benefit assessment, the consent form should be reviewed and updated if necessary. All subjects (including those already being treated) should be informed of the new information and must give their written informed consent to continue in the study.

If the general practitioner is informed of his patients’ participation in the clinical study, this should be mentioned in the consent form.

10.3 Insurance coverage

The insurance coverage (study subject insurance) laid down in § 40 AMG is in force. For each subject, the Sponsor has taken out insurance.

Proft F, *et al. Ann Rheum Dis* 2024; 83:599–607. doi: 10.1136/ard-2023-224699

11 INDEPENDENT ETHICS COMMITTEE AND REGULATORY AUTHORITIES

11.1 Approval of the Study by Regulatory Authorities and Independent Ethics Committees

According to §§ 40-42 of the AMG it is the responsibility of the Sponsor to obtain and maintain independent approval from the federal regulatory authority (BfArM/PEI) and a positive opinion from the competent ethics committees to conduct the study.

The written approval of the Central Independent Ethics Committee of the „Landesamt für Gesundheit und Soziales, Ethik-Kommission des Landes Berlin" will be obtained prior to the enrolment of patients.

Furthermore, the commencement of the study together with the study protocol and the decision of the Central Ethics Committee involved will be reported to the PEI (Paul-Ehrlich-Institut, Langen, Germany) and the locally responsible Ethics Committees and local supervisory authorities.

11.2 Notification of the Study

According to § 67 German drug law (AMG) the sponsor or the sponsor's designee is responsible to notify competent regional authority and the federal regulatory authority about the study and all principal investigators of the participating investigational sites. If no other agreements are made, the sponsor or the sponsor's designee will take over responsibility for investigator's obligation to report (§ 12 (3) GCP-V).

11.3 Report and Documentation Obligation

The sponsor or the sponsor's designee is responsible to comply with the report and documentation obligation according to § 13 GCP-V.

The investigator is responsible to comply with the report and documentation obligation according to § 12 GCP-V.

Proft F, *et al. Ann Rheum Dis* 2024; 83:599–607. doi: 10.1136/ard-2023-224699

12 CONDITIONS FOR MODIFYING THE PROTOCOL

Protocol modifications to on-going studies must be made via protocol amendment. The sponsor is responsible to obtain independent approval for the amendment from the federal regulatory authority (PEI) and a positive opinion from the competent ethics committees if required according to § 10 GCP-V.

According to § 67 AMG amendments competent regional authorities and the federal regulatory authority must be notified about the amendment, if they concern items according to § 12 Abs. 1 GCP-V.

Proft F, *et al. Ann Rheum Dis* 2024; 83:599–607. doi: 10.1136/ard-2023-224699

13 STATISTICAL ANALYSIS PLAN

The study consists of a 12-week run-in period, 96-week core period (randomized treatment) and 4-week follow-up period. The primary efficacy endpoint will be the absolute progression of the mSASSS score over two years of therapy in both treatment groups. The primary analysis will be based on the mean of the mSASSS scores evaluated by 2 trained readers for the spinal x-rays performed at screening and at Week 108 in the ITT population.

A database lock will occur for the purposes of the efficacy and safety analysis, once all data up to week 112 have been collected and cleaned.

Study biostatistician: Dr. Joachim Listing, German Rheumatism Research Center (Deutsches Rheumaforschungszentrum – DRFZ, Berlin).

13.1 Definition of Population for Analysis

Primary analysis will be based on the ITT population. A secondary analysis will be based on all patients with complete sets of radiographs. For the purpose of safety analyses all patients receiving at least one dose of study drug (starting at Visit 2) will be included.

13.2 Intent-to-Treat Population

Intent-to-treat population (ITT) is defined to include all subjects who received at least one dose of study medication in phase II of the study (starting from Visit 6) and where the primary variable was measured at least once under study medication.

13.3 Per Protocol Population

Per protocol population is defined as a subset of the ITT population who completed the study without any major protocol violations. Specific reasons for warranting exclusion will be agreed and documented in a statistical analysis plan prior to the closing of the database. Not all protocol deviators and violators will be excluded from the per protocol population.

13.4 Safety Population

The safety population is defined to include all subjects who received at least one dose of the trial medication, whether withdrawn prematurely or not.

Proft F, *et al. Ann Rheum Dis* 2024; 83:599–607. doi: 10.1136/ard-2023-224699

13.5 Efficacy analyses

The primary analysis will be based on all patients who entered phase II of the trial (ITT population). Multiple imputation methods with mSASSS score at baseline as covariate (SAS procedure PROC MI) will be applied to deal with missing radiographs in the primary analysis. The Mann-Whitney test will be used to compare the primary outcome (change in the mSASSS score) between the treatment groups. Cumulative probability plots of will be built to visualise the finding.

In a secondary analysis the Mann-Whitney test will be used to compare radiographic progression in the subgroup of patients with complete sets of radiographs. A likelihood approach will be applied to deal with missing data in secondary outcome parameters assessed at multiple time points (Molenberghs G., Kenward M.G. Missing data in clinical studies. Chichester: John Wiley & Sons; 2007). For that reason linear mixed models (SAS procedure PROC MIXED) will be applied to compare means of secondary outcome parameters over time. Time and group, (time*group) will be included in these models as fixed effects and patient-ID as a random parameter (included in PROC MIXED via the repeated statement with type = un for an unstructured covariance matrix). In the case of skewed data PROC MIXED will be applied to log-transformed parameter (CRP) or non-parametric tests (Mann-Whitney test, non-parametric analysis of covariance) will be applied (e.g., for biomarker data). Spearman rank correlation and non-parametric analysis of covariance will furthermore be used to investigate the association between radiographic progression and biomarkers of inflammation and bone/cartilage turnover (Bathke A, Brunner E. A nonparametric alternative to analysis of covariance. In: Akritas MG, Politis DN eds. Recent advantages and trends in nonparametric statistics. Amsterdam: Elsevier Science and Technology 2003: 109-20).

Nonresponder imputation for missing response data and chi-square tests will additionally be applied to compare response rates. Two-sided p values < 0.05 will be considered to be statistically significant.

13.6 Safety Data Analysis

Safety data will be analysed describing frequency and types of adverse events. All adverse events will be coded and tabulated by body system and preferred term for individual events within each body system, and will be presented in descending frequency. Adverse events will also be tabulated by severity and relationship to the study medication. Serious adverse events will be summarized separately.

Proft F, *et al. Ann Rheum Dis* 2024; 83:599–607. doi: 10.1136/ard-2023-224699

13.7 Methods against Bias

There will be three different methods avoiding possible bias:

1) Randomization at Week 12: subjects who present a good response to TNF-blocker therapy will be randomized 1:1 by a FDA 21 part 11 compliant software tool (SecuTrial) of the Coordinating Center for Clinical Studies of the Charité Berlin (KKS Koordinierungszentrum für Klinische Studien) in order to receive Golimumab plus continuous treatment with Celecoxib vs. Golimumab alone. This study protocol allows the inclusion of patients in whom the baseline radiographic examination of the spine was done up to two years prior to screening. A possible bias caused by differences in the length of the time intervals between the baseline and the week 108 radiographic examination of the patients will therefore be controlled for by randomization. Prior to randomization patients will be stratified by time period between radiographic examination used as baseline radiographic examination and the visit at week 12. Three months intervals (0-3, 4-6 etc. months) will be considered to build up these strata. Block randomization stratified by these strata will be used. This method will therefore balance the treatment groups for an important factor with the potential to influence the outcome. Of note, only subjects who are eligible for randomization represent the target population of the study.
2) Blinded and independent reading of the spinal radiographs: the primary outcome (radiographic progression) will be assessed centrally by two trained readers blinded to all data including treatment allocation and also to the time points the x-rays were taken.
3) A possible bias caused by patients with missing radiographs at follow up (e.g. dropouts) is considered in the statistical analysis (see 13.5).

13.8 Sample Size and Power Calculation

The study is designed for a total sample size of n =170 subjects who will enrol in the phase I of the study. The power calculations are based on the findings of Kroon et al[16 43], our own results from GESPIC Cohort[18], and those of Braun et al[23]. We assume a worsening in the mSASSS score of 1.7±2.8 in patients of the control group and of 0.2±1.6 in patients with continuous NSAIDs intake. We considered such a difference of 1.5 mSASSS units as clinically relevant and planned the sample size of this study by means of a two-sided (α=0.05) Welch-Satterthwaite t-test accordingly. A smaller treatment effect, which was

observed in a trial comparing diclofenac continuously vs. on demand and which would be missed by our trial design, would be considered as clinically not relevant. To detect the difference with an 80% power, the sample size of n = 38 in each group is needed in the phase II of the trail. This is also true if the Mann-Whitney test is applied. Since in the case of skewed non-normal distributions (like the mSASSS) the power of the Mann-Whitney-test is not lower than that of the t-test even in the case of ties (Rasch D et al, 2007). However, the power will decrease by the application of multiple imputations to deal with missing radiographs of dropouts in the ITT population. We estimated this decrease by: a) an estimation of dropout rates and b) the use of own mSASSS data. Considering that all subjects enrolled in the phase II will be responders to Golimumab therapy receiving this same treatment for the whole period of the trial, along with our experience in conducting randomized controlled trials, we presume dropout rates of less than 20% during phase II. For this reason and based on our own mSASSS data, we expect an increase in the variance of mSASSS progression because of multiple imputation by less than 25%. Considering this possible increase, a sample size of n = 100 patients is needed for phase II of the trial to detect the expected and clinically relevant difference of an average 1.5 mSASSS points with an 80% power in the ITT population. In addition we assumed that 60% of the subjects enrolled in phase I of the study (n = 100 out of 170) would be eligible for randomization and participation in phase II (based on the rate of the BASDAI response of at least 2 absolute points in AS patients with elevated CRP).

14 QUALITY CONTROL AND QUALITY ASSURANCE / MONITORING

The sponsor performs quality control and assurance checks on all clinical studies that it sponsors. Before enrolling any subjects in this study, sponsor personnel and the investigator review the protocol, the CRF's and instructions for their completion, the procedure for obtaining informed consent, and the procedure for reporting AE's and serious AE's. Quality control and quality assurance will be provided by an external monitor who will visit all participating study sites by regular intervals. During these site visits information recorded in the CRF's is verified against source documents.

The principal investigator will review all serious events (SAE) and state whether they are severe unexpected serious adverse reactions (SUSAR) according to GCP guidelines.

14.1 Monitoring

The monitor has the responsibility to familiarize the investigator(s) and the entire centre staff involved in the study with all study procedures including the administration of study drug.

The sponsor must provide a trained monitor to assist the investigator(s) in conducting the clinical study. The monitor must visit the clinical study centre on a regular basis and at least before the first subject has been enrolled, once during the course of the study, and at study completion. The monitor has the responsibility of reviewing the on-going study with the investigator(s) to verify adherence to the protocol and to deal with any problems that arise. At all times the sponsor must maintain the confidentiality of the study documents. It is the responsibility of the study monitor to verify the study documents against the subject's original medical records.

The investigator (or his/her deputy) agrees to co-operate with the monitor to ensure that any problems detected in the course of these monitoring visits are resolved.

14.2 Study Documentation, CRFs and Record Keeping

The investigator will permit study related monitoring, audits, institutional review board IRB / IEC review, and regulatory inspections by providing direct access to source data and documents.

Proft F, *et al. Ann Rheum Dis* 2024; 83:599–607. doi: 10.1136/ard-2023-224699

All information will be recorded on source documents. All required data will be recorded in the CRF's. All CRF data must be submitted to the sponsor throughout and at the end of the study.

If an investigator retires, relocates, or otherwise withdraws from conducting the study, the investigator must notify the sponsor to agree upon an acceptable storage solution. Regulatory agencies will be notified with the appropriate documentation.

The CRF data are stored in a database and processed electronically. The sponsor medical monitor reviews the data for safety information. The data is reviewed for legibility, completeness, and logical consistency. Requests for data clarification are forwarded to the investigative site for resolution.

By signing this protocol the investigator agrees that the sponsor or sponsor's designee, IRB/IEC, or Regulatory Agency representatives may consult and/or copy study documents in order to verify CRF data. By signing the consent form, the subject agrees to this process. If study documents will be photocopied during this process of verification, the subject will be identified by unique code only; full name/initials will be masked.

It is understood by the investigator that the information and data included in this protocol may be disclosed to and used by the investigator's staff and associates as may be necessary to conduct this clinical study.

The information derived from this clinical study will be used by the sponsor and therefore, may be disclosed by the sponsor as required to other clinical investigators or other government agencies. In order to allow for the use of the information derived from this clinical study, it is understood by the investigator that there is an obligation to provide the sponsor with complete test results and all data from this clinical study.

14.3 Investigator's Files/Retention of Documents

The investigator must maintain adequate and accurate records to enable the conduct of the study to be fully documented and the study data to be subsequently verified. These documents should be classified into two different separate categories: Investigator's Study File, and subject/patient data.

The Investigator's Study File will contain all essential documents as the protocol/amendments, Case Report and Query Forms, patient information and informed consent form, Ethics Committee and federal regulatory authority approval, notification of the federal regulatory authority and competent regional authorities, drug accountability records,

Proft F, *et al. Ann Rheum Dis* 2024; 83:599–607. doi: 10.1136/ard-2023-224699

staff curriculum vitae and authorization forms and other appropriate documents/correspondence.

Patient data include patient hospital/clinic records (medical records, pathology and laboratory reports, x-ray, etc.) and signed informed consent forms and subject screening and eligibility forms.

The investigator shall retain and preserve one copy of all data generated in the course of the study, specifically including but not limited to those documents defined by GCP as essential documents, for at least 10 years after the end of the study or for a longer period if required by the applicable regulatory requirement(s) or the funder of the trial.

If the investigator cannot guarantee this archiving requirement at the investigational site for any or all of the documents, special arrangements must be made between the investigator and the sponsor to store these in a sealed container(s) outside of the site so that they can be returned sealed to the investigator in case of a regulatory audit. If source documents are required for the continued care of the patient, appropriate copies should be made for storing outside of the site.

The sponsor must archive the protocol, documentation, approvals and all other essential documents related to the study, including certificates that satisfactory audit and inspection procedures have been carried out, as long as the test product(s) remains on the market.

All documents must be archived in a secure place and treated as confidential material.

14.4 Source Documents and Background Data

The investigator shall supply the sponsor on request with any required background data from the study documentation or clinic records. This is particularly important when CRF are illegible or when errors in data transcription are suspected. In case of special problems and/or governmental queries or requests for audit inspections, it is also necessary to have access to the complete study records, provided that patient confidentiality is protected. According to the standards of the data protection law, all data obtained in the course of a clinical study must be treated with discretion in order to guarantee the rights of the patient's privacy.

Proft F, *et al. Ann Rheum Dis* 2024; 83:599–607. doi: 10.1136/ard-2023-224699

14.5 Audits and Inspections

This study may be audited by the sponsor, any person authorized by the sponsor or the competent health authority in order to determine the authenticity of the recorded data and compliance with the study protocol.

The investigator should understand that source documents for this trial should be available to appropriately qualified personnel from the sponsor/monitors/auditor/health authority inspectors after appropriate notification needed for source data verification and proper review of the study progress. The verification of the CRF data must be by direct inspection of source documents. The investigator agrees to comply with the Sponsor and regulatory authority requirements regarding the auditing of the study.

All material used in clinical studies are subjected to quality control.

14.6 Case Report Forms

For each patient enrolled, an electronic CRF must be completed and (electronically) verified or signed by the principal investigator or authorized delegate from the study staff. If a patient withdraws from the study, the reason must be noted on the CRF. If a patient is withdrawn from the study because of a treatment-limiting adverse event, thorough efforts should be made to clearly document the outcome.

All forms should be typed or filled out using indelible ink, and must be legible. Errors should be crossed out but not obliterated, the correction inserted, and the change initialled and dated by the investigator or his/her authorized delegate. The investigator should ensure the accuracy, completeness, legibility, and timeliness of the data reported to the sponsor in the CRFs and in all required reports.

Proft F, *et al. Ann Rheum Dis* 2024; 83:599–607. doi: 10.1136/ard-2023-224699

15 CONFIDENTIALITY OF TRIAL DOCUMENTS AND SUBJECT RECORDS

The investigator and the sponsor (or designee) must assure that according to the standards of the data protection law, all data obtained in the course of a clinical study must be treated with discretion in order to guarantee the rights of the patient's privacy. CRFs or other documents should be submitted to the sponsor in a pseudonym manner. Each patient will receive a unique trial number, patient’s initials or date of birth should not be used for the pseudonymisation. The investigator should keep a patient identification log showing codes and names strictly confidential.

Proft F, *et al. Ann Rheum Dis* 2024; 83:599–607. doi: 10.1136/ard-2023-224699

16 REFERENCES

1. van der Linden S, Valkenburg HA, Cats A. Evaluation of diagnostic criteria for ankylosing spondylitis. A proposal for modification of the New York criteria. Arthritis Rheum 1984;**27**(4):361-8.
2. Creemers MC, Franssen MJ, van't Hof MA, et al. Assessment of outcome in ankylosing spondylitis: an extended radiographic scoring system. Ann Rheum Dis 2005;**64**(1):127-9.
3. Braun J, Sieper J. Ankylosing spondylitis. Lancet 2007;**369**(9570):1379-90.
4. Landewe R, Dougados M, Mielants H, et al. Physical function in ankylosing spondylitis is independently determined by both disease activity and radiographic damage of the spine. Ann Rheum Dis 2009;**68**(6):863-7.
5. Feldtkeller E, Bruckel J, Khan MA. Scientific contributions of ankylosing spondylitis patient advocacy groups. Curr Opin Rheumatol 2000;**12**(4):239-47.
6. Braun J, Brandt J, Listing J, et al. Treatment of active ankylosing spondylitis with infliximab: a randomised controlled multicentre trial. Lancet 2002;**359**(9313):1187-93.
7. Franke LC, Ament AJ, van de Laar MA, et al. Cost-of-illness of rheumatoid arthritis and ankylosing spondylitis. Clin Exp Rheumatol 2009;**27**(4 Suppl 55):S118-23.
8. Braun J, van den Berg R, Baraliakos X, et al. 2010 update of the ASAS/EULAR recommendations for the management of ankylosing spondylitis. Ann Rheum Dis 2011;**70**(6):896-904.
9. Braun J, Zochling J, Baraliakos X, et al. Efficacy of sulfasalazine in patients with inflammatory back pain due to undifferentiated spondyloarthritis and early ankylosing spondylitis: a multicentre randomised controlled trial. Ann Rheum Dis 2006;**65**(9):1147-53.
10. Haibel H, Rudwaleit M, Braun J, et al. Six months open label trial of leflunomide in active ankylosing spondylitis. Ann Rheum Dis 2005;**64**(1):124-6.

11. Haibel H, Brandt HC, Song IH, et al. No efficacy of subcutaneous methotrexate in active ankylosing spondylitis: a 16-week open-label trial. Ann Rheum Dis 2007;**66**(3):419-21.
12. van der Heijde D, Sieper J, Maksymowych WP, et al. 2010 Update of the international ASAS recommendations for the use of anti-TNF agents in patients with axial spondyloarthritis. Ann Rheum Dis 2011;**70**(6):905-8.
13. Baeten D, Sieper J, Braun J, et al. Secukinumab, an Interleukin-17A Inhibitor, in Ankylosing Spondylitis. N Engl J Med 2015;**373**(26):2534-48.
14. Wanders A, Heijde D, Landewe R, et al. Nonsteroidal antiinflammatory drugs reduce radiographic progression in patients with ankylosing spondylitis: a randomized clinical trial. Arthritis Rheum 2005;**52**(6):1756-65.
15. Yoon DS, Yoo JH, Kim YH, et al. The effects of COX-2 inhibitor during osteogenic differentiation of bone marrow-derived human mesenchymal stem cells. Stem cells and development 2010;**19**(10):1523-33.
16. Kroon F, Landewe R, Dougados M, et al. Continuous NSAID use reverts the effects of inflammation on radiographic progression in patients with ankylosing spondylitis. Ann Rheum Dis 2012;**71**(10):1623-9.
17. Poddubnyy D, Haibel H, Listing J, et al. Baseline radiographic damage, elevated acute-phase reactant levels, and cigarette smoking status predict spinal radiographic progression in early axial spondylarthritis. Arthritis Rheum 2012;**64**(5):1388-98.
18. Poddubnyy D, Rudwaleit M, Haibel H, et al. Effect of non-steroidal anti-inflammatory drugs on radiographic spinal progression in patients with axial spondyloarthritis: results from the German Spondyloarthritis Inception Cohort. Ann Rheum Dis 2012;**71**(10):1616-22.
19. Sieper J, Listing J, Poddubnyy D, et al. Effect of continuous versus on-demand treatment of ankylosing spondylitis with diclofenac over 2 years on radiographic progression of the spine: results from a randomised multicentre trial (ENRADAS). Ann Rheum Dis 2015.

20. van der Heijde D, Landewe R, Einstein S, et al. Radiographic progression of ankylosing spondylitis after up to two years of treatment with etanercept. Arthritis Rheum 2008;**58**(5):1324-31.

21. van der Heijde D, Landewe R, Baraliakos X, et al. Radiographic findings following two years of infliximab therapy in patients with ankylosing spondylitis. Arthritis Rheum 2008;**58**(10):3063-70.

22. van der Heijde D, Salonen D, Weissman BN, et al. Assessment of radiographic progression in the spines of patients with ankylosing spondylitis treated with adalimumab for up to 2 years. Arthritis Res Ther 2009;**11**(4):R127.

23. Braun J, Baraliakos X, Hermann KG, et al. The effect of two golimumab doses on radiographic progression in ankylosing spondylitis: results through 4 years of the GO-RAISE trial. Ann Rheum Dis 2014;**73**(6):1107-13.

24. Chang JK, Li CJ, Liao HJ, et al. Anti-inflammatory drugs suppress proliferation and induce apoptosis through altering expressions of cell cycle regulators and pro-apoptotic factors in cultured human osteoblasts. Toxicology 2009;**258**(2-3):148-56.

25. Tegeder I, Pfeilschifter J, Geisslinger G. Cyclooxygenase-independent actions of cyclooxygenase inhibitors. FASEB journal : official publication of the Federation of American Societies for Experimental Biology 2001;**15**(12):2057-72.

26. Dougados M, Simon P, Braun J, et al. ASAS recommendations for collecting, analysing and reporting NSAID intake in clinical trials/epidemiological studies in axial spondyloarthritis. Ann Rheum Dis 2011;**70**(2):249-51.

27. Jenkinson TR, Mallorie PA, Whitelock HC, et al. Defining spinal mobility in ankylosing spondylitis (AS). The Bath AS Metrology Index. J Rheumatol 1994;**21**(9):1694-8.

28. Sieper J, Rudwaleit M, Baraliakos X, et al. The Assessment of SpondyloArthritis international Society (ASAS) handbook: a guide to assess spondyloarthritis. Ann Rheum Dis 2009;**68 Suppl 2**:ii1-44.

29. Garrett S, Jenkinson T, Kennedy LG, et al. A new approach to defining disease status in ankylosing spondylitis: the Bath Ankylosing Spondylitis Disease Activity Index. J Rheumatol 1994;**21**(12):2286-91.
30. Calin A, Garrett S, Whitelock H, et al. A new approach to defining functional ability in ankylosing spondylitis: the development of the Bath Ankylosing Spondylitis Functional Index. J Rheumatol 1994;**21**(12):2281-5.
31. Kiltz U, van der Heijde D, Boonen A, et al. Development of a health index in patients with ankylosing spondylitis (ASAS HI): final result of a global initiative based on the ICF guided by ASAS. Ann Rheum Dis 2015;**74**(5):830-5.
32. Wendel-Vos GC, Schuit AJ, Saris WH, et al. Reproducibility and relative validity of the short questionnaire to assess health-enhancing physical activity. J Clin Epidemiol 2003;**56**(12):1163-9.
33. Kvien TK, Heiberg T, Hagen KB. Minimal clinically important improvement/difference (MCII/MCID) and patient acceptable symptom state (PASS): what do these concepts mean? Ann Rheum Dis 2007;**66 Suppl 3**:iii40-1.
34. Zochling J, van der Heijde D, Burgos-Vargas R, et al. ASAS/EULAR recommendations for the management of ankylosing spondylitis. Ann Rheum Dis 2006;**65**(4):442-52.
35. Song IH, Poddubnyy DA, Rudwaleit M, et al. Benefits and risks of ankylosing spondylitis treatment with nonsteroidal antiinflammatory drugs. Arthritis Rheum 2008;**58**(4):929-38.
36. Bakland G, Gran JT, Nossent JC. Increased mortality in ankylosing spondylitis is related to disease activity. Ann Rheum Dis 2011;**70**(11):1921-5.
37. Haroon NN, Paterson JM, Li P, et al. Patients With Ankylosing Spondylitis Have Increased Cardiovascular and Cerebrovascular Mortality: A Population-Based Study. Ann Intern Med 2015;**163**(6):409-16.

Proft F, *et al. Ann Rheum Dis* 2024; 83:599–607. doi: 10.1136/ard-2023-224699

38. Maneiro JR, Salgado E, Gomez-Reino JJ. Immunogenicity of monoclonal antibodies against tumor necrosis factor used in chronic immune-mediated Inflammatory conditions: systematic review and meta-analysis. JAMA internal medicine 2013;**173**(15):1416-28.
39. Braun J, Deodhar A, Inman RD, et al. Golimumab administered subcutaneously every 4 weeks in ankylosing spondylitis: 104-week results of the GO-RAISE study. Ann Rheum Dis 2012;**71**(5):661-7.
40. Deodhar A, Braun J, Inman RD, et al. Golimumab administered subcutaneously every 4 weeks in ankylosing spondylitis: 5-year results of the GO-RAISE study. Ann Rheum Dis 2015;**74**(4):757-61.
41. Sieper J, van der Heijde D, Dougados M, et al. A Randomized, Double-blind, Placebo-controlled, 16-Week Study of Subcutaneous Golimumab in Patients With Active Non-radiographic Axial Spondyloarthritis. Arthritis Rheumatol 2015.
42. Kiltz U, Sieper J, Kellner H, et al. [German Society for Rheumatology S3 guidelines on axial spondyloarthritis including Bechterew's disease and early forms: 8.4 Pharmaceutical therapy, 8.5 Evaluation of therapy success of pharmaceutical measures]. Z Rheumatol 2014;**73 Suppl 2**:78-96.
43. Kroon F, Landewe R, Dougados M, et al. Continuous NSAID use reverts the effects of inflammation on radiographic progression in patients with ankylosing spondylitis. Ann Rheum Dis 2012.

17 SIGNATURE PAGE

Study title: “**CO**mparison of the effect of treatment with **N**SAIDs added to anti-TNF therapy versus anti-TNF therapy alone on progression of **S**tr**U**ctural damage in the spine over two years in patients with anky**L**osing spondylitis: a randomized controlled multicentre trial **(CONSUL)”**

Protocol number: CONSUL2016
Protocol version: 1.3 including Amendment No.1
Date: 13.02.2017

Coordinating Investigator & Designee of the Sponsor:

Prof. Dr. Denis Poddubnyy
Medizinische Klinik für Gastroenterologie,
Infektiologie und Rheumatologie
Campus Benjamin Franklin
Charité Universitätsmedizin Berlin

______________________________ ______________________________
Signature Date

Proft F, *et al. Ann Rheum Dis* 2024; 83:599–607. doi: 10.1136/ard-2023-224699
