# Supplementary material for: Comparison of the effect of treatment with NSAIDs added to anti-TNF therapy versus anti-TNF therapy alone on the progression of structural damage in the spine over 2 years in patients with radiographic axial spondyloarthritis from the randomised-controlled CONSUL trial
Source: Ann Rheum Dis. 2024 Jan 16;83(5):599–607. doi: 10.1136/ard-2023-224699 (PMC11041582; doi:10.1136/ard-2023-224699)
Supplement: Supplementary data [file ard-2023-224699supp003.pdf]

SUPPLEMENTARY MATERIAL:

Supplementary Figure 1. Cumulative probability plot of mSASSS change in patients with high risk of radiographic progression in the CONSUL study: A) 65 patients with elevated CRP values (> 5mg/L) at baseline, B) 54 patients with syndesmophyte(s) at baseline, and C) 30 patients with both elevated CRP AND syndesmophyte(s) at baseline.

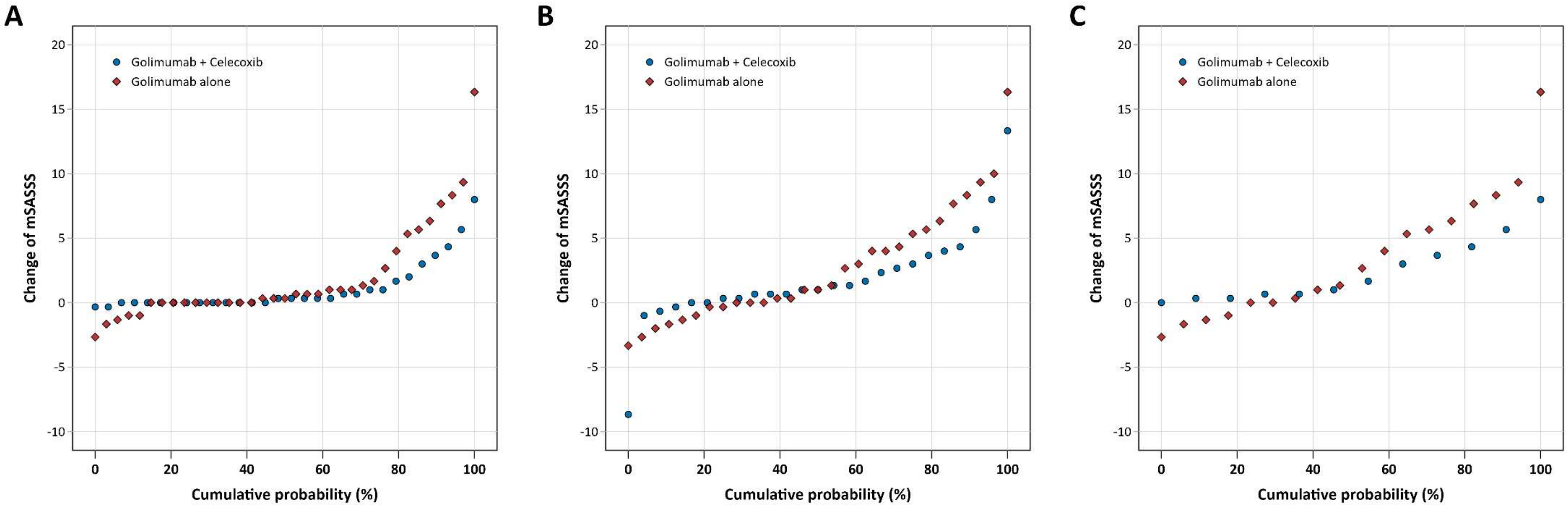

Supplementary Figure 2. Clinical outcome measures during the core phase of the CONSUL study.

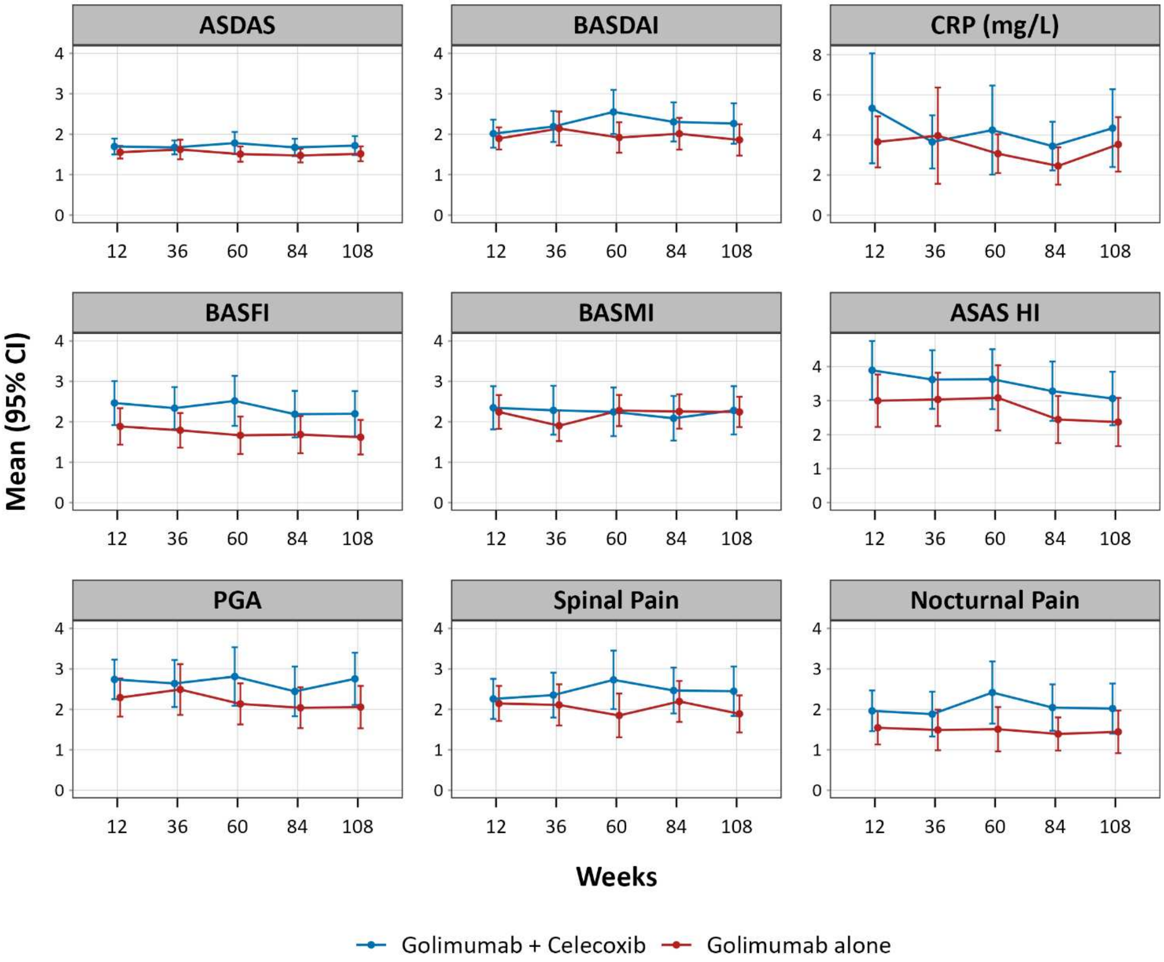

ASDAS, Axial Spondyloarthritis Disease Activity Index; BASDAI, Bath Ankylosing Spondylitis Disease Activity Index; BASFI, Bath Ankylosing Spondylitis Functional Index; BASMI, Bath Ankylosing Spondylitis Metrology Index; CRP, c-reactive protein; PGA, Patient Global Assessment.

Supplementary table 1. Multivariable linear regression analyses for the association between change in disease activity parameters and treatment groups.

Model*	Outcome	Comparison	Beta (95% CI)	P Value
1	Change in BASDAI	Monotherapy vs Combination	0.06 (-0.44, 0.86)	0.52
2	Change in BASDAI	Monotherapy vs Combination	0.01 (-0.64, 0.69)	0.94
3	Change in BASDAI	Monotherapy vs Combination	0.00 (-0.55, 0.55)	0.99
4	Change in BASDAI	Monotherapy vs Combination	-0.03 (-0.72, 0.49)	0.71
5	Change in ASDAS	Monotherapy vs Combination	0.07 (-0.17, 0.45)	0.37
6	Change in ASDAS	Monotherapy vs Combination	0.03 (-0.22, 0.36)	0.65

*All multivariable models adjusted for duration of biologic therapy before baseline, smoking status, and HLA-B27 positivity. Following parameters were added to the adjustment sets of corresponding model:
Model 1: BASDAI at baseline,
Model 2: ASDAS at baseline,
Model 3: Age, sex, symptom duration, CRP at baseline, and BASDAI at baseline,
Model 4: Age, sex, symptom duration, and ASDAS at baseline,
Model 5: ASDAS at baseline,
Model 6: Age, sex, symptom duration, and ASDAS at baseline.
ASDAS, Axial Spondyloarthritis Disease Activity Index; BASDAI, Bath Ankylosing Spondylitis Disease Activity Index; CRP, c-reactive protein
